# Supplementary material for: Transcriptional firing represses bactericidal activity in cystic fibrosis airway neutrophils
Source: Cell Rep Med. 2021 Apr 8;2(4):100239. doi: 10.1016/j.xcrm.2021.100239 (PMC8080108; doi:10.1016/j.xcrm.2021.100239)
Supplement: Document S1. Tables S1 and S3 and Figures S1–S5 [file mmc1.pdf]

Cell Reports Medicine, Volume 2

Supplemental information

Transcriptional firing represses bactericidal activity in cystic fibrosis airway neutrophils

Camilla Margaroli, Diego Moncada-Giraldo, Dalia Arafat Gulick, Brian Dobosh, Vincent D. Giacalone, Osric A. Forrest, Fangxu Sun, Chunhui Gu, Amit Gaggar, Haydn Kissick, Ronghu Wu, Greg Gibson, and Rabindra Tirouvanziam

Cell Reports Medicine – Research Article

TRANSCRIPTIONAL FIRING REPRESSES BACTERICIDAL ACTIVITY IN CYSTIC FIBROSIS AIRWAY NEUTROPHILS

Camilla Margaroli, Diego Moncada-Giraldo, Dalia Arafat Gulick,

Brian Dobosh, Vincent D. Giacalone, Osric A. Forrest, Fangxu Sun, Chunhui Gu,

Amit Gaggar, Haydn Kissick, Ronghu Wu, Greg Gibson, Rabindra Tirouvanziam

Supplemental information

Tables 3

Figures 5

CF patients In vitro/RNASeq	Age	Gender	Ethnicity	CFTR genotype	Modulator Therapy
1	30	F	Caucasian	HO	Tezacaftor / Ivacaftor
2	24	M	Caucasian	HZ	None
3	37	M	Caucasian	HO	Tezacaftor / Ivacaftor
4	29	M	Caucasian	HO	None
5	22	F	Caucasian	OT	Tezacaftor / Ivacaftor
6	31	F	Caucasian	HZ	None
7	32	F	Asian	OT	None
**CF patients Microarrays**					
1	50	M	Caucasian	HO	None
2	32	F	Caucasian	HZ	None
3	12	F	Caucasian	HO	None
4	25	M	Caucasian	HO	None
5	32	M	Caucasian	OT	None
6	38	F	Caucasian	HZ	None
7	32	M	Caucasian	HO	None
**COPD patients**					
1	56	F	Caucasian	N/A	None
2	55	M	Caucasian	N/A	None
3	45	F	Caucasian	N/A	None
**Healthy donors**					
1	27	F	Caucasian	N/A	None
2	24	M	Caucasian	N/A	None
3	25	F	Caucasian	N/A	None
4	23	M	Caucasian	N/A	None
5	40	M	Caucasian	N/A	None

Table S1. Patient demographics related to STAR Methods. Legend: F, female; HO, homozygous for F508Del mutation; HZ, heterozygous for one F508Del mutation and another CFTR mutation; M, male; N/A, not available; OT, other mutations than F508Del.

		1 hour		2 hours		4 hours		6 hours	
Gene set name	Genes in gene set (K)	Genes in overlap (K)	FDR q-value	Genes in overlap (K)	FDR q-value	Genes in overlap (K)	FDR q-value	Genes in overlap (K)	FDR q-value
Gene expression transcription (R)	1486	19	0.0076	161	6.01E-16	0	NA	101	4.58E-45
Generic transcription pathway (R)	1218	17	0.0076	142	1.55E-16	0	NA	83	1.68E-36
Interferon gamma response (H)	200	0	NA	52	2.80E-19	35	1.64E-14	35	3.58E-28
Cytokine signaling in immune system (R)	856	0	NA	114	3.37E-17	59	6.89E-07	102	1.00E-68
Allograft rejection (H)	200	0	NA	39	2.47E-10	21	9.33E-05	31	2.95E-23
Adaptive immune system (R)	811	0	NA	117	5.83E-20	66	9.52E-11	93	8.96E-61
Innate immune system (R)	1104	0	NA	138	1.74E-18	111	3.52E-26	157	1.82E-122
Interferon alpha response (H)	97	0	NA	28	1.66E-11	19	2.46E-08	0	NA
Interferon signaling (R)	202	0	NA	38	1.13E-09	25	6.28E-07	0	NA
Signaling by interleukins (R)	631	0	NA	78	4.96E-10	0	NA	73	3.34E-47
Hemostasis (R)	674	0	NA	0	NA	50	9.51E-07	65	6.49E-37
Neutrophil degranulation (R)	478	0	NA	0	NA	64	6.48E-21	92	7.61E-81
Immunoregulatory interactions between a lymphoid and a non-lymphoid cell (R)	186	0	NA	0	NA	23	1.87E-06	28	1.15E-20
Generation of 2nd messenger molecules (R)	37	0	NA	15	2.24E-08	0	NA	0	NA
Heme metabolism (H)	200	0	NA	35	3.76E-08	0	NA	0	NA
Intracellular signaling by 2nd messengers (R)	302	0	NA	47	3.97E-09	0	NA	0	NA
Toll like receptor cascades (R)	153	0	NA	30	3.77E-08	0	NA	0	NA
MAPK signaling pathway (K)	267	0	NA	45	8.03E-10	0	NA	0	NA
T cell receptor signaling pathway (K)	108	0	NA	32	1.36E-13	0	NA	0	NA
Metabolism of lipids (R)	738	0	NA	84	3.97E-09	0	NA	0	NA
MAPK pathway (R)	81	0	NA	22	1.47E-08	0	NA	0	NA

Vesicle mediated transport (R)	723	0	NA	80	3.56E-08	0	NA	0	NA
Chromatin organization (R)	272	0	NA	44	4.14E-09	0	NA	0	NA
Signaling by Rho GTPases (R)	450	0	NA	0	NA	33	2.10E-04	0	NA
Platelet homeostasis (R)	86	0	NA	0	NA	13	1.71E-04	0	NA
Beta catenin independent Wnt signaling (R)	145	0	NA	0	NA	17	1.71E-04	0	NA
Interferon alpha beta signaling (R)	70	0	NA	0	NA	12	1.25E-04	0	NA
Leishmania infection (K)	72	0	NA	0	NA	13	3.25E-05	0	NA
Fc gamma R mediated phagocytosis (K)	96	0	NA	0	NA	14	1.25E-04	0	NA
ESR mediated signaling (R)	219	0	NA	0	NA	23	3.25E-05	0	NA
Signaling by Notch (R)	244	0	NA	0	NA	23	1.51E-04	0	NA
Transport of small molecules (R)	728	0	NA	0	NA	48	4.62E-05	0	NA
Signaling by Wnt (R)	327	0	NA	0	NA	27	2.09E-04	0	NA
Complement (H)	200	0	NA	0	NA	0	NA	36	2.00E-29
Regulation of actin cytoskeleton (K)	213	0	NA	0	NA	0	NA	30	2.80E-21
Inflammatory response (H)	200	0	NA	0	NA	0	NA	31	2.95E-23
Signaling by receptor tyrosine kinases (R)	468	0	NA	0	NA	0	NA	48	3.94E-28
Chemokine signaling pathway (K)	189	0	NA	0	NA	0	NA	30	8.06E-23
TNFa signaling via NFkB (H)	200	0	NA	0	NA	0	NA	28	8.34E-20
Other interleukin signaling (R)	288	0	NA	0	NA	0	NA	32	1.24E-19
Class I MHC mediated antigen processing presentation (R)	370	0	NA	0	NA	0	NA	37	3.76E-21

Table S3. Kinetics of pathway downregulation in CFASN-migrated neutrophils (related to Figure 3 and Figure S6). The transcriptome of neutrophils was analyzed at 1, 2, 4, and 6 hours post-transmigration towards CFASN. Pathway enrichment analysis was conducted by GSEA on downregulated genes at each time point compared to blood (FDR q-value <5%). Legend: H, hallmarks; K, Kegg; R, reactome.

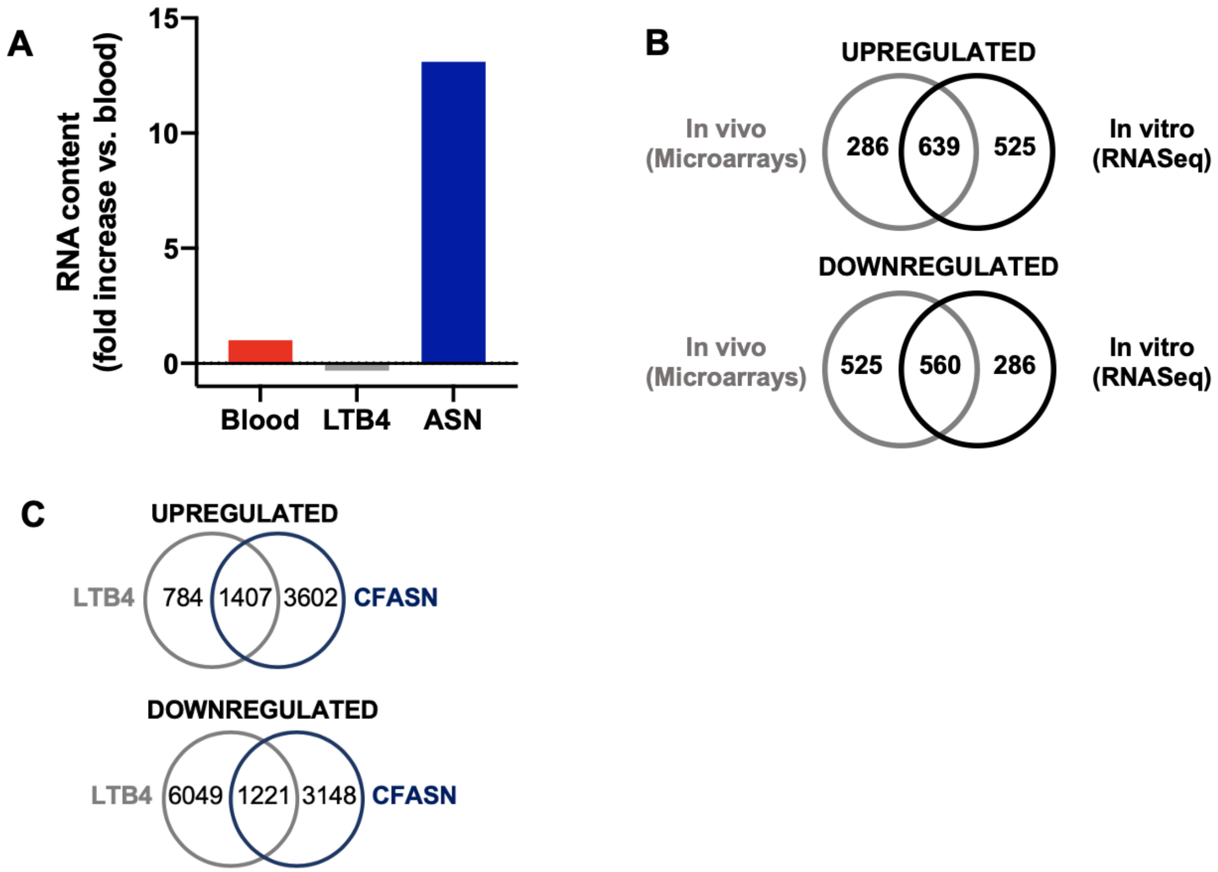

Figure S1 related to Figure 1. GRIM neutrophils show an increase in RNA content in the cystic fibrosis lung *in vivo* and *in vitro*. (A) Blood neutrophils from CF patients (red, N=1) were transmigrated (TM) *in vitro* using sputum supernatant (CFASN, blue) or the chemoattractant leukotriene B4 (LTB4) as a transmigration control (grey). Total RNA quantification was obtained using the bioanalyzer. **(B)** Genes that were commonly upregulated or downregulated both *in vivo* (microarrays) and *in vitro* (RNASeq) compared to blood. **(C)** Differential gene expression of upregulated and downregulated genes was determined comparing transmigrated conditions to blood. Gene expression regulation was determined by log2 gene expression greater than 2 and smaller than -2.

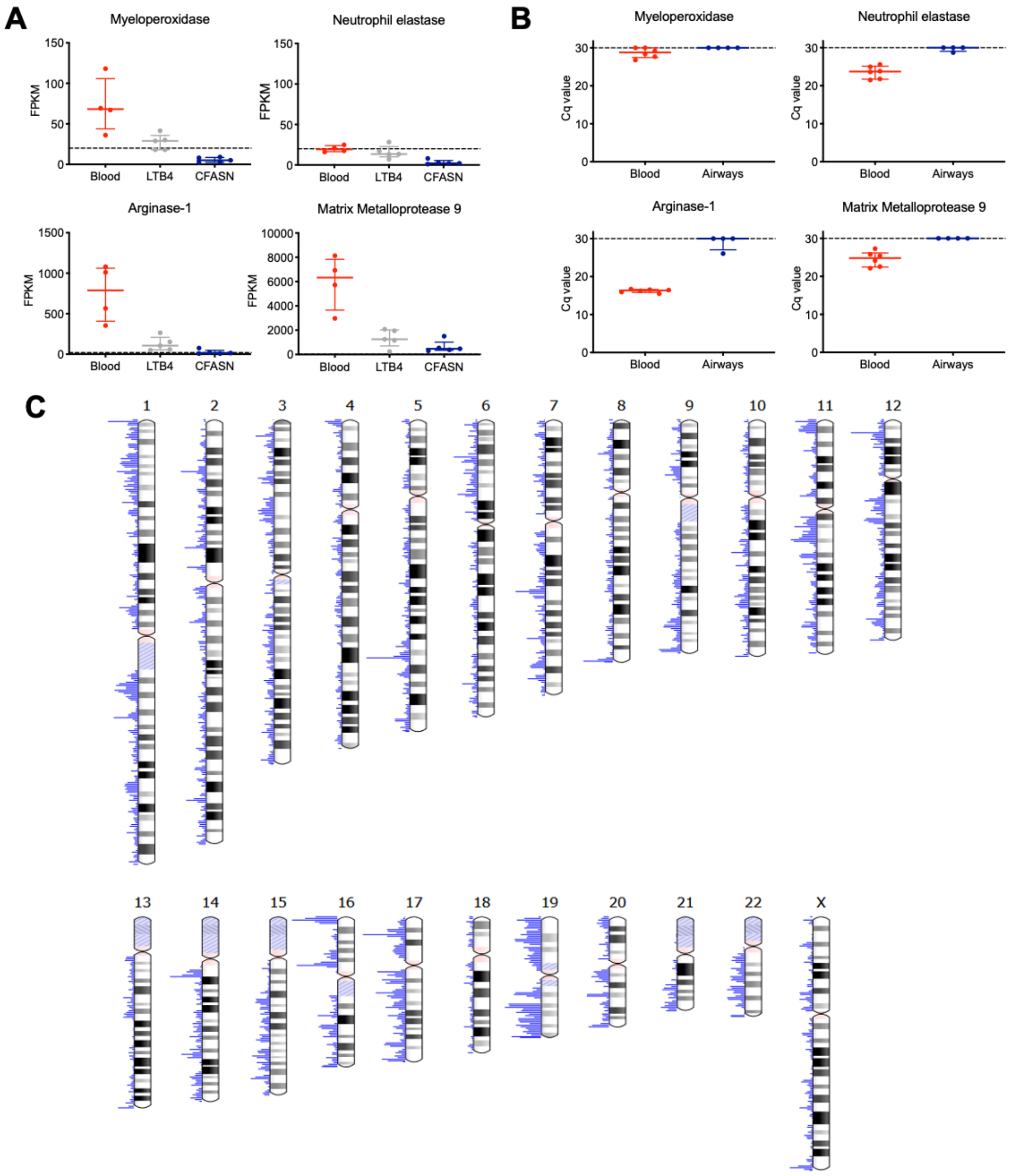

Figure S2 related to figure 2. CF airway neutrophils lack *de novo* expression of main granule effector proteins. RNA of effector granule proteins measured by RNASeq *in vitro* **(A)** and by Fluidigm *in vivo* **(B)** are not detectable in CF airway GRIM neutrophils. Dotted line represents lower limit of detection (20 FPKM for RNASeq, and 30 Ct for Fluidigm). **(C)** Histograms represent RNA counts of transcripts detected by RNASeq in neutrophils transmigrated to CFASN at their respective chromosomal positions showing the breadth of transcriptional firing.

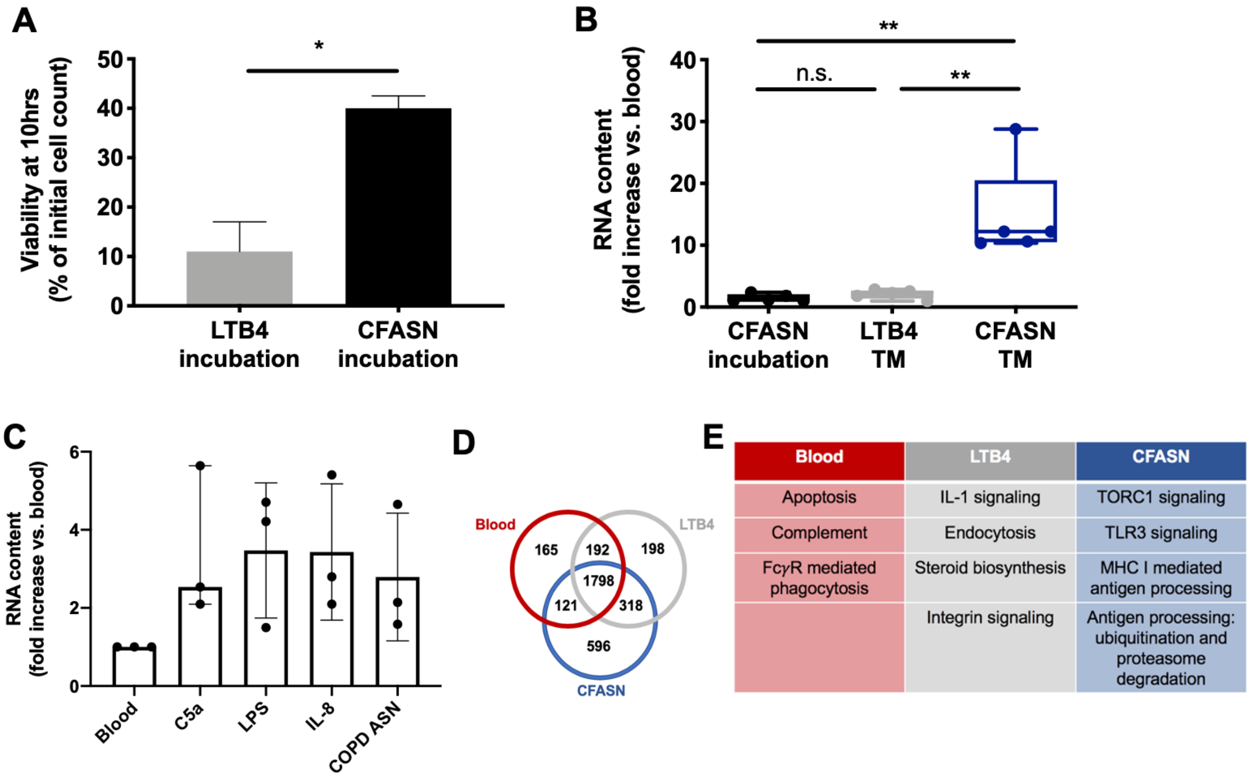

Figure S3 related to Figure 2. Incubation in CFASN increases neutrophil lifespan, but fails to recapitulate the transcriptional burst. Neutrophils isolated from blood of healthy donors were incubated in LTB4 or CFASN for 10 hours and analyzed for viability **(A)**. Neutrophils incubated in CFASN, or transmigrated in LTB4 or CFASN **(B)**, or C5a, LPS, IL-8 or COPD ASN **(C)** for 10 hours were analyzed for their total RNA content. Data are shown as median and interquartile range and were analyzed using the Wilcoxon matched-pairs signed rank test, *p<0.05, **p<0.01. **(D)** Blood and neutrophils transmigrated *in vitro* for 10 hours (N=3) were lysed and the extracted proteins were analyzed by LC-MS untargeted proteomics. Proteins present in at least two repeats were used to generate the Venn diagram, while unique proteins for each condition were used to determine pathway enrichment by GSEA .**(E)** Pathways listed showed an FDR less than 5% and a p-value less than 0.01.

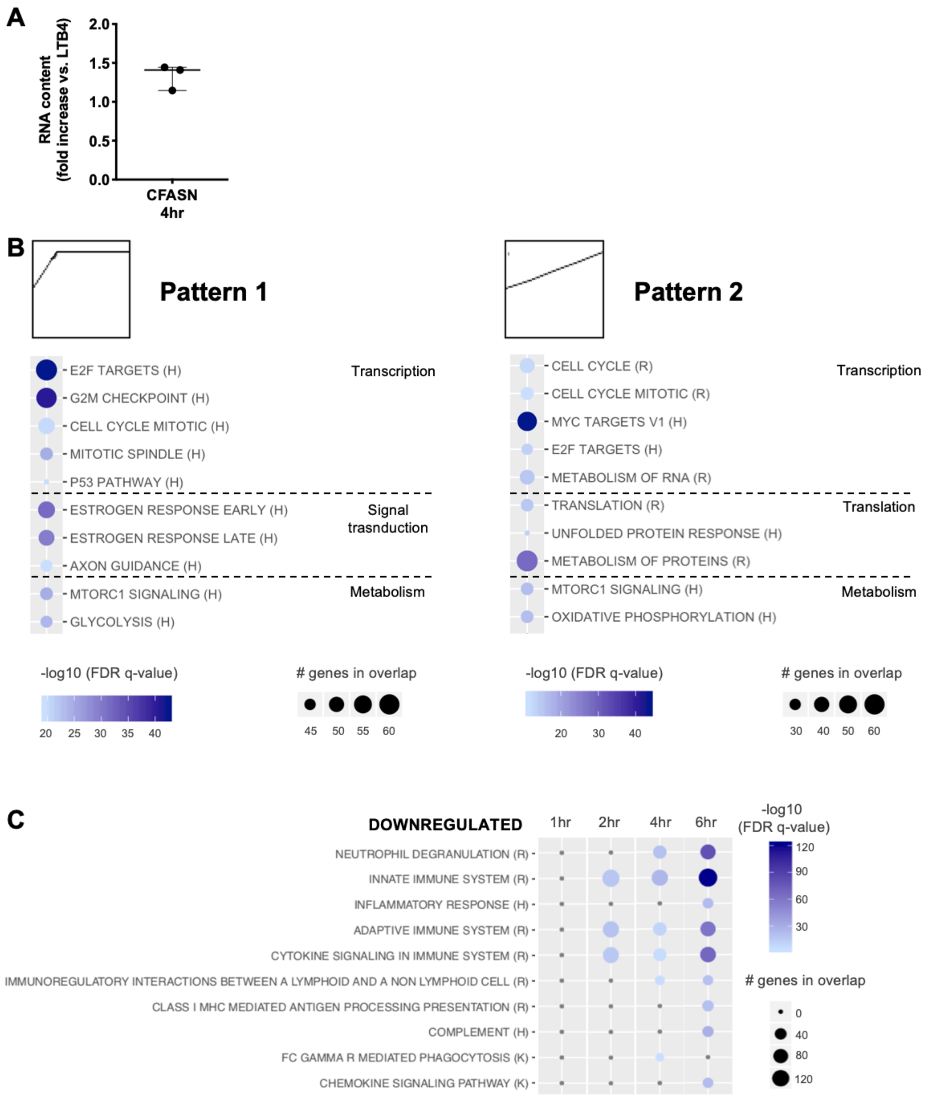

Figure S4 related to Figure 3. Pathway analysis of the GRIM phenotype development. Neutrophils were transmigrated towards CFASN and their transcriptome was analyzed at 1, 2, 4, and 6 hours post-transmigration compared to matched blood (N=3). (**A**) Comparison of total RNA content, measured by bioanalyzer, between CFASN and LTB4 transmigrated neutrophils at 4hr post-transmigration. Upregulated genes were analyzed by STEM and two patterns of gene upregulation over time were identified **(B)**. Pathway enrichment analysis was performed by GSEA (FDR q-value <5%) for upregulated **(B)** and downregulated genes **(C)**.

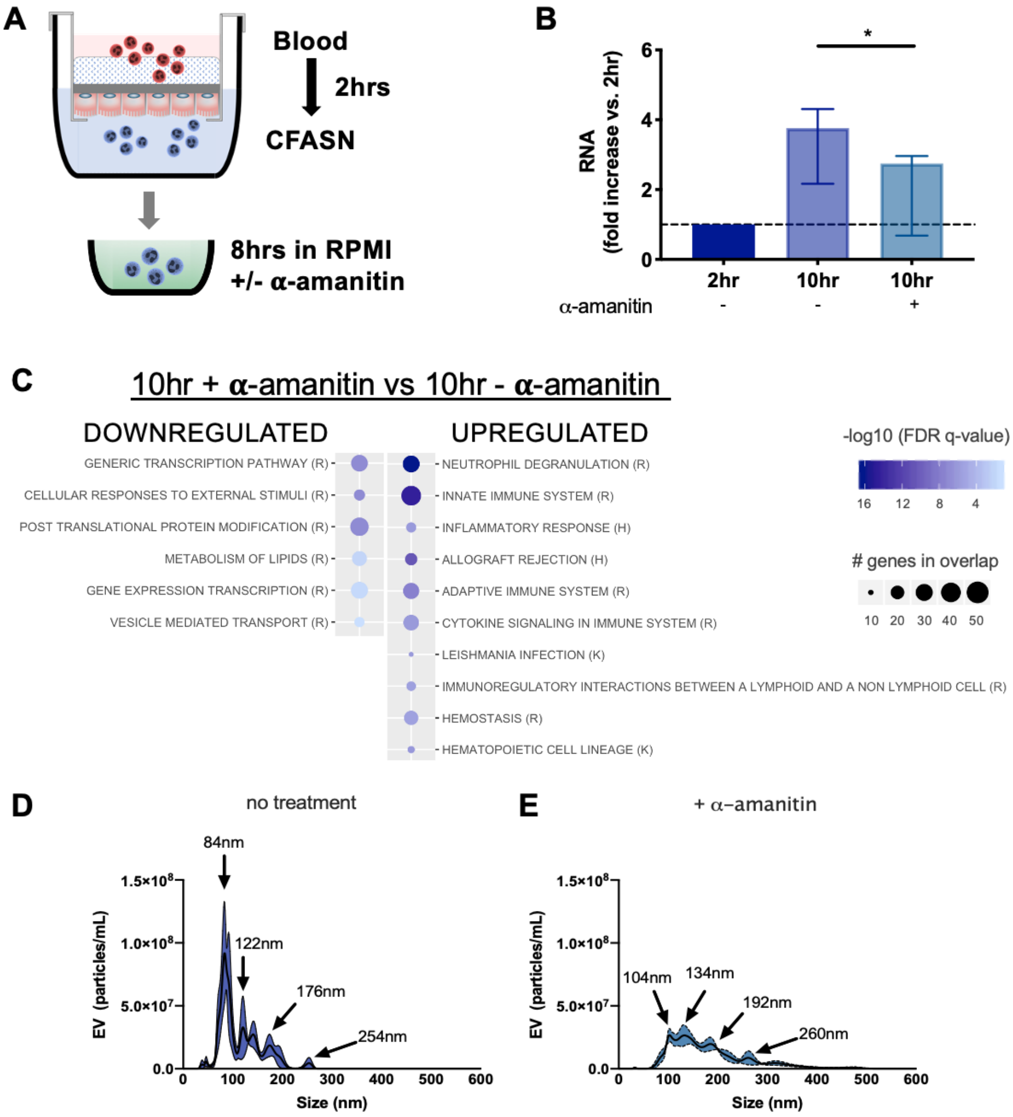

Figure S5 related to Figure 4. Blockade of *de novo* transcription modulates airway neutrophil phenotype in the CF microenvironment. (A) Neutrophils were transmigrated *in vitro* for 2 hours towards CFASN and subsequently incubated for 8 hours with or without the transcriptional blocker α-amanitin. CFASN transmigrated neutrophils treated with α-amanitin showed reduced amounts of total RNA content **(B)**. **(C)** Comparison of neutrophil treated with the transcriptional blocker to those not treated showed a shift in their transcriptional profile upon treatment. Downregulated and upregulated genes analyzed by GSEA using hallmark (H), reactome (R), and Kegg (K) datasets. Data are shown as median and interquartile range and were analyzed using the Wilcoxon matched-pairs signed rank test, *p<0.05. Extracellular vesicle concentration and size were determined using the Nanosight NS3000 for neutrophil not treated **(D)** and treated **(E)** with α-amanitin.
