# Supplementary material for: Transcriptional firing represses bactericidal activity in cystic fibrosis airway neutrophils
Source: Cell Rep Med. 2021 Apr 8;2(4):100239. doi: 10.1016/j.xcrm.2021.100239 (PMC8080108; doi:10.1016/j.xcrm.2021.100239)
Supplement: Document S2. Article plus supplemental information [file mmc3.pdf]

Cell Reports
Medicine

Article

Transcriptional firing represses bactericidal activity in cystic fibrosis airway neutrophils

Graphical abstract

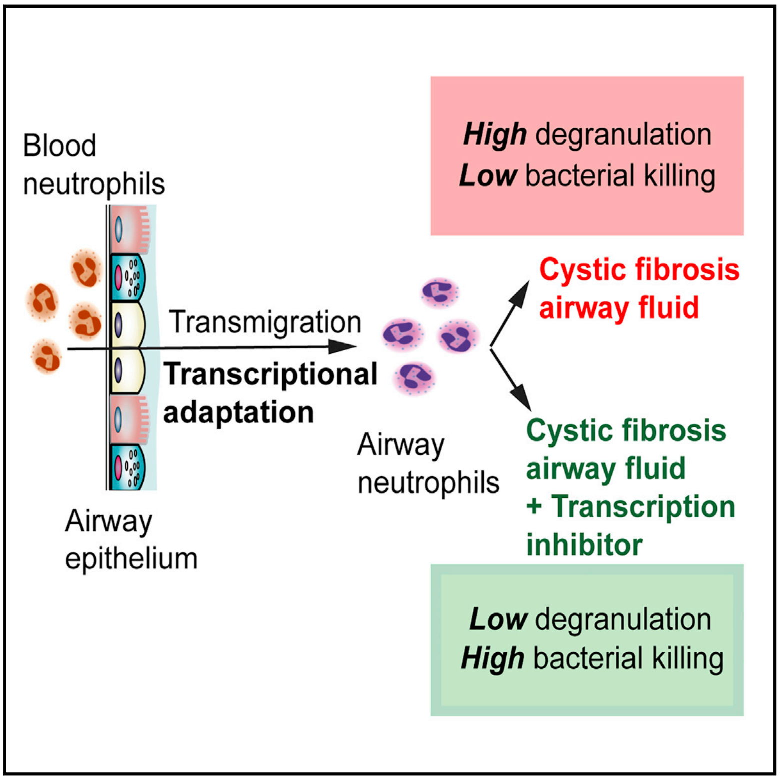

Highlights

- Neutrophils recruited to cystic fibrosis airways undergo *de novo* RNA transcription
- Neutrophil adaptation to CF airways leads to downregulation of their antimicrobial genes
- Transcriptional blockade restores CF airway neutrophil bactericidal capacity

Authors

Camilla Margaroli,
Diego Moncada-Giraldo,
Dalia Arafat Gulick, ..., Ronghu Wu,
Greg Gibson, Rabindra Tirouvanziam

Correspondence

tirouvanziam@emory.edu

In brief

Neutrophils are often considered terminally differentiated and poised for bacterial killing. Here, Margaroli et al. show that human neutrophils are transcriptionally and translationally plastic after migration into the cystic fibrosis lung, leading to adaptations that can affect even canonical functions (i.e., bacterial clearance).

Margaroli et al., 2021, Cell Reports Medicine *2*, 100239
April 20, 2021 © 2021 The Author(s).
https://doi.org/10.1016/j.xcrm.2021.100239

CellPress

Cell Reports Medicine

CellPress
OPEN ACCESS

Article

Transcriptional firing represses bactericidal activity in cystic fibrosis airway neutrophils

Camilla Margaroli,[1,2] Diego Moncada-Giraldo,[1,2] Dalia Arafat Gulick,[3] Brian Dobosh,[1,2] Vincent D. Giacalone,[1,2] Osric A. Forrest,[1,2] Fangxu Sun,[4] Chunhui Gu,[1,2] Amit Gaggar,[5,6] Haydn Kissick,[7] Ronghu Wu,[4] Greg Gibson,[3] and Rabindra Tirouvanziam[1,2,8,*]

[1]Department of Pediatrics, Emory University School of Medicine, Atlanta, GA, USA
[2]Center for CF & Airways Disease Research, Children's Healthcare of Atlanta, Atlanta, GA, USA
[3]School of Biological Sciences, Georgia Institute of Technology, Atlanta, GA, USA
[4]Department of Chemistry & Biochemistry, Georgia Institute of Technology, Atlanta, GA, USA
[5]Department of Medicine, Division of Pulmonary, Allergy & Critical Care Medicine, University of Alabama at Birmingham, Birmingham, AL, USA
[6]Birmingham VA Medical Center, Birmingham, AL, USA
[7]Department of Urology, Emory University School of Medicine, Atlanta, GA, USA
[8]Lead contact
*Correspondence: tirouvanziam@emory.edu
https://doi.org/10.1016/j.xcrm.2021.100239

SUMMARY

Neutrophils are often considered terminally differentiated and poised for bacterial killing. In chronic diseases such as cystic fibrosis (CF), an unexplained paradox pits massive neutrophil presence against prolonged bacterial infections. Here, we show that neutrophils recruited to CF airways *in vivo* and in an *in vitro* transmigration model display rapid and broad transcriptional firing, leading to an upregulation of anabolic genes and a downregulation of antimicrobial genes. Newly transcribed RNAs are mirrored by the appearance of corresponding proteins, confirming active translation in these cells. Treatment by the RNA polymerase II and III inhibitor α-amanitin restores the expression of key antimicrobial genes and increases the bactericidal capacity of CF airway neutrophils *in vitro* and in short-term sputum cultures *ex vivo*. Broadly, our findings show that neutrophil plasticity is regulated at the site of inflammation via RNA and protein synthesis, leading to adaptations that affect their canonical functions (i.e., bacterial clearance).

INTRODUCTION

Postnatal neutrophil development occurs in the bone marrow over 10–14 days,[1] with cells of the neutrophilic lineage progressing from progenitor to mitotic and post-mitotic stages.[2] In humans, five stages of neutrophil differentiation are identified that depend on the wave-like expression of specific transcription factors controlling the production of antimicrobial receptors and effector proteins that are sequentially packaged into three granule subsets and secretory vesicles.[1,3–6] This differentiation is accompanied by progressive chromatin condensation, which culminates in the formation of mature neutrophils that are released into the bloodstream, featuring a characteristic multilobulated nucleus.

Upon the presence of inflammatory stimuli, neutrophil interaction with soluble factors, such as cytokines and danger- or pathogen-associated molecular patterns, as well as migration into tissues, induces neutrophil priming, a pre-activated state reflected by specific phenotypic and functional changes.[7,8] These changes are mediated by intracellular signaling pathways and physical adaptation dictated by the passage through the tissue, both of which may lead to the modification of the chromatin state in neutrophils and the transcription of new genes.[9,10]

Chronic respiratory diseases are one of the main causes of morbidity and mortality worldwide,[11,12] and are often characterized by unresolving neutrophilic inflammation. Among these diseases, cystic fibrosis (CF) represents a prototypical example of sustained inflammation driven by lifelong recruitment of neutrophils from the blood into the airway lumen, progressive connective tissue damage by neutrophil enzymes, and fatal loss of lung function. Previously, we established that neutrophil recruitment to CF airways is followed by profound phenotypic and functional adaptations dictated by the local microenvironment. We dubbed this adaptive fate GRIM, because it is characterized by primary granule release,[13–16] immunomodulatory activities toward other immune subsets,[17] reduced bacterial clearance,[18] and metabolic licensing.[19,20] The latter is reflected by an increased expression of nutrient transporters, enhanced glycolytic capacity, increased number of polyribosomes, and sustained activation of the mechanistic target of rapamycin (mTOR) anabolic pathway, a master regulator of cellular metabolism and nucleotide synthesis.[21–23] Despite this sustained activation, CF airway neutrophils are unable to contain bacteria that progressively

This is an open access article under the CC BY license (http://creativecommons.org/licenses/by/4.0/).

colonize patients' lungs and worsen their outcomes. The mechanisms underlying adaptive changes in CF airway neutrophils and their lack of bactericidal activity remain poorly understood. Here, we investigated whether these adaptive changes were dependent upon *de novo* transcription and translation.

RESULTS

Airway neutrophils display profound transcriptional changes *in vivo* and *in vitro*

The recruitment of neutrophils into inflamed CF airways induces profound changes in their phenotypic and functional profile, called the GRIM fate,[18] which includes metabolic adaptation to the airway microenvironment.[19,20] To determine whether the acquisition of the GRIM fate was concomitant with changes in the RNA profile, blood and airway (sputum) neutrophils from seven adult patients with CF (Table S1) were analyzed by flow cytometry (Figure 1A). CF airway neutrophils displayed the characteristic primary granule hyperexocytosis, reflected by increased surface CD63 expression, and loss of the phagocytic receptor CD16. In addition to these previously described changes, airway neutrophils showed a median 3.5-fold increase in total RNA content compared to blood neutrophils (Figure 1B).

To study this phenomenon further, we used a transmigration *in vitro* model that recapitulates the GRIM fate.[18] In this model, naive blood neutrophils are migrated through a small airway epithelium grown at the air-liquid interface toward CF airway fluid supernatant (CFASN, which corresponds to sputum sequentially centrifuged to remove cells and bacteria) or leukotriene B4 (LTB4, transmigration control), placed apically (Figure 1C). Similar to our *in vivo* observations in CF sputum samples and as previously shown in our model,[18] healthy donor neutrophils transmigrated *in vitro* to CFASN showed increased surface CD63 and decreased surface CD16 expression, as well as increased total RNA content (>10-fold) (Figures 1D and 1E). Meanwhile, neutrophils transmigrated to LTB4 did not differ from blood neutrophils, except for a 2-fold increase in total RNA content. Similar modulations of RNA content were observed in CF blood neutrophils transmigrated *in vitro*, suggesting that the origin of the airway fluid, but not that of the blood neutrophils, is a major influence on their behavior upon recruitment to the airways (Figure S1A).

Next, to establish whether *in vitro* induction of CF airway neutrophils induces transcriptional differentiation, we compared the fold change between airway and blood neutrophils. Among the 2,010 genes that showed a differential change in CF airway compared to blood neutrophils *in vivo*, 639 genes were also found upregulated *in vitro*, while 560 were downregulated both *in vivo* and *in vitro* (Figure S1B). The transcriptional profile of airway neutrophils, analyzed using the genes that showed the same regulation *in vivo* and *in vitro*, mirrored the functional adaptation previously observed in these cells (Figure 1F). Genes coding for anabolic pathways and production of new proteins were upregulated compared to matched blood neutrophils, whereas canonical antimicrobial and cell death pathways were downregulated, suggesting that *de novo* transcription and translation are involved in the pathological conditioning of neutrophils by the CF airway microenvironment.

The CF airway microenvironment imprints a unique transcriptional and proteomic profile onto recruited neutrophils

To better define the changes observed in CFASN-transmigrated neutrophils, we performed both targeted and untargeted analyses of the transcriptome obtained from blood neutrophils pre-transmigration, as well as CFASN- and LTB4-transmigrated neutrophils. These three conditions were completely discriminated by principal component analysis (PCA) (Figure 2A), and comparison of the CFASN- and LTB4-transmigrated neutrophils showed considerable overlap in genes that were up- or downregulated by at least by 4-fold (|log2| >2) compared to blood (Figure S1C). Of those, 1,407 genes were upregulated and 1,221 were downregulated in both LTB4 and CFASN conditions. However, a distinctive profile was observed in CFASN-transmigrated neutrophils, with 3,602 and 3,148 uniquely upregulated and downregulated genes, respectively (Figure S1C).

Next, we investigated phenotypic changes defined as hallmarks of CF lung disease and the GRIM fate of CF airway neutrophils. Of those, the active release of primary granules and resulting increased extracellular presence of neutrophil elastase (NE) and myeloperoxidase (MPO) are among the best correlates of lung function and structural damage in pediatric and adult CF patients.[16,24–26] In addition to increased extracellular NE, we previously observed an increase in cell-associated NE in CF airway GRIM neutrophils compared to matched blood neutrophils *in vivo.*[14] Therefore, we investigated whether GRIM neutrophils were producing NE and other effector proteins through *de novo* transcription by cross-referencing our results with those obtained in a prior comprehensive proteomic study of human neutrophil granules.[27] Most of the upregulated transcripts in CFASN-transmigrated versus blood neutrophils coded for proteins related to the structure, transport, and docking of granules to their target compartment (Table S2), while most of the granule effector proteins were downregulated (Figure 2B).

To confirm these comparative results, we determined whether the transcripts of downregulated effector proteins were present at all in neutrophils transmigrated to CFASN and LTB4 *in vitro* (Figure S2A). Apart from matrix metalloproteinase 9 (MMP9), other effector proteins such as NE, MPO, and arginase-I had transcripts that were below the detection limit. To confirm these results with samples collected *in vivo*, we quantified the levels of the same transcripts by multiplex qPCR (Fluidigm) in blood and airway neutrophils sorted from patients. Similar to our *in vitro* results, the levels of NE, MPO, and arginase-I were below the detection limit in CF airway GRIM neutrophils *in vivo* (Figure S2B). These findings suggest that the pathological accumulation of primary granule effector proteins in the airway fluid of CF patients and within airway neutrophils is likely not caused by *de novo* transcription, but rather by the uptake of proteins discharged by waves of GRIM neutrophils and accumulated in the airway lumen over time.

Neutrophils recruited to CF airways revert to an anabolic state with active transcription and translation

The above findings suggest that the transcriptional burst seen in neutrophils transmigrated to CFASN is regionally controlled, rather than dependent upon a global switch from

CellPress
OPEN ACCESS

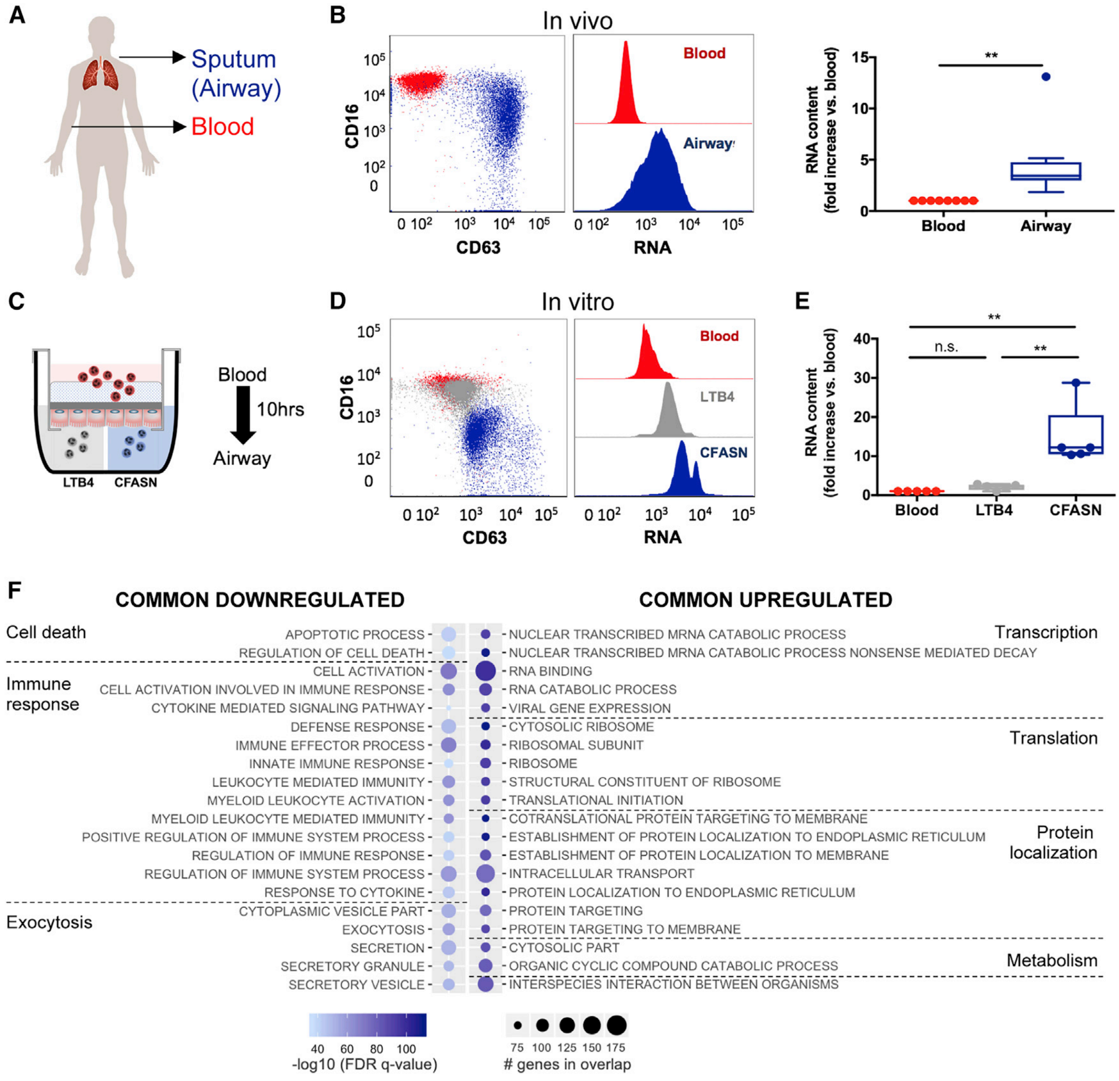

Figure 1. GRIM neutrophils show an increase in RNA content in the cystic fibrosis lung *in vivo* and *in vitro*

(A) Blood (red) and sputum (blue) neutrophils from patients with CF (N = 7) were analyzed by flow cytometry.

(B) Airway neutrophils displayed a characteristic loss of the phagocytic receptor CD16 and release of primary granules, measured by surface CD63. Increased total RNA content compared to their matched counterpart was measured by flow cytometry.

(C) Blood neutrophils (red, N = 5) were transmigrated *in vitro* to sputum supernatant from patients with CF (CFASN, blue) or the chemoattractant leukotriene B4 (LTB4) as a transmigration control (gray).

(D and E) Flow cytometry analysis revealed an increase in RNA content in CFASN airway neutrophils compared to matched blood neutrophils, which was confirmed by quantification by bioanalyzer (E).

(F) Genes that were commonly upregulated or downregulated both *in vivo* and *in vitro* were used to conduct a Gene Ontology (GO) term RNA analysis. Top 20 pathways for the upregulated and downregulated genes are shown; significance of enriched pathways was established using the false discovery rate (FDR) q < 5%.

Results are shown as median and interquartile range. Statistical analysis was performed by Wilcoxon matched-pairs signed rank test; **p < 0.01, n.s., not significant.

heterochromatin to euchromatin. This observation prompted us to investigate the breadth of transcriptional burst observed in CFASN neutrophils, which revealed activity across all chromosomes (Figure S2C). To distinguish the relative effects on the acquisition of the GRIM fate by neutrophils of (1) transepithelial migration and (2) conditioning by CFASN, we focused our analysis on the comparison between CFASN incubation and transmigration and LTB4 and CFASN transmigration conditions. Incubation in CFASN increased the neutrophil lifespan compared to those incubated in LTB4 (Figure S3A), suggesting that the microenvironment in the CF airways could delay neutrophil apoptosis. However, incubation in CFASN (Figure S3B) or transmigration toward chronic obstructive pulmonary disease (COPD) ASN (Figure S3C) failed to induce the

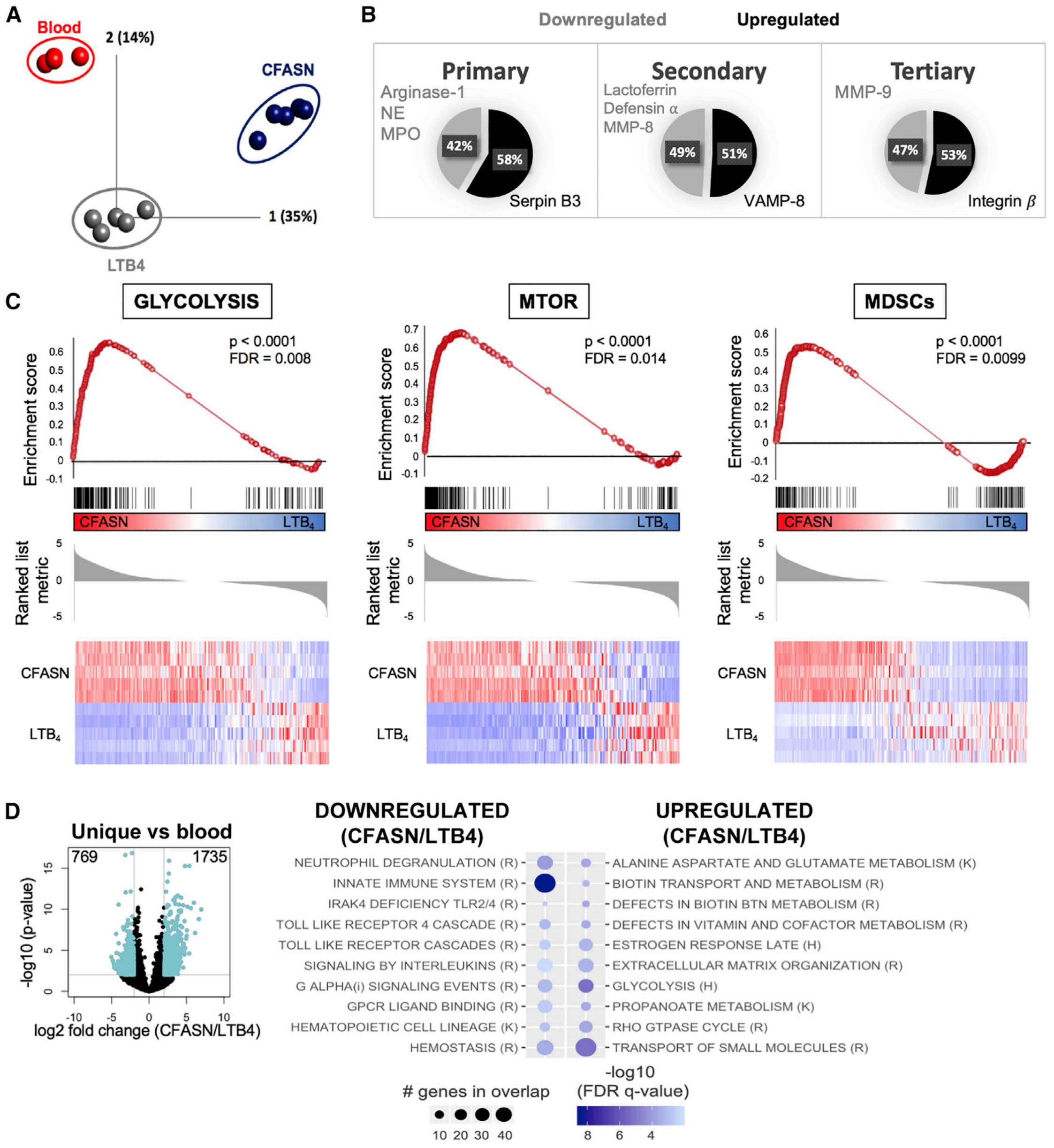

Figure 2. The CF airway microenvironment imparts distinct transcriptional signatures onto GRIM neutrophils

(A) Principal component analysis (PCA) of the transcriptome from neutrophils used in the *in vitro* transmigration model showed a distinct profile for each condition (blood, LTB4-transmigrated, and CFASN-transmigrated; N = 5 independent experiments).

(B) Primary, secondary, and tertiary granule effector proteins were downregulated (gray) in the CFASN group compared to their blood counterpart, while respective structural granule proteins were upregulated (black).

(C) Gene set enrichment analysis (GSEA) showed enriched glycolysis and mTOR metabolic pathways in the CFASN neutrophils compared to LTB4, as well as changes in the immunological profile, reflecting the acquisition of myeloid-derived suppressor cell (MDSC) function.

(D) Of the genes uniquely upregulated or downregulated compared to the blood, those that were significantly different (log2 fold change greater or less than 2 or −2, and $p < 0.01$) between CFASN and LTB4 were analyzed by GSEA using the hallmark (H), reactome (R), and KEGG (K) datasets. Top 10 pathways (FDR < 5%) are shown for the upregulated and downregulated gene sets.

full transcriptional burst observed in neutrophils transmigrated toward CFASN, pointing toward a "two-hit" mechanism requiring both transmigration and subsequent exposure to the CF airway milieu to fully alter the behavior of lung-recruited neutrophils.

Of the total transcript pool, 3,417 and 1,731 genes were significantly upregulated and downregulated, respectively, in neutrophils transmigrated in CFASN compared to LTB4. Moreover, metabolic and immunological changes previously described at the protein and functional levels were mirrored in the

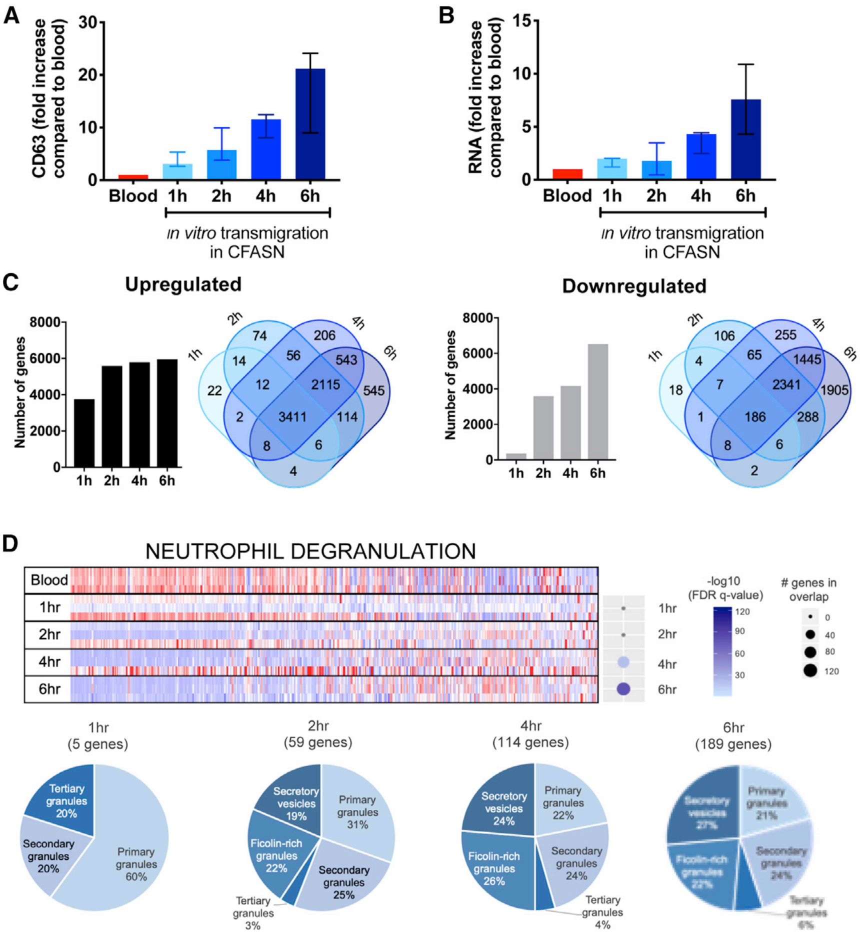

Figure 3. Transcriptional and functional adaptation of neutrophils to the CF airway microenvironment occurs over time

Blood neutrophils (red) were transmigrated *in vitro* toward CFASN and collected at 1, 2, 4, and 6 h post-transmigration (N = 5 independent experiments).

(A and B) Surface CD63 expression was quantified by flow cytometry (A), and RNA content by bioanalyzer (B).

(C) The transcriptional profile obtained by RNA sequencing for each time point was compared to blood (N = 3 subjects). Significantly different genes are defined as log2 greater than 2 or less than −2 for upregulated genes (left) and dowregulated genes (right), respectively, and indicated in Venn diagram form to show the overlap between time points.

(D) Pathway analysis for downregulated genes by GSEA (FDR q < 5%) shows reduced expression of granule effector proteins (reactome "neutrophil degranulation" pathway) over time relative to blood. Dot plots indicate the number of genes in the overlap between the experimental gene set and the reactome neutrophil degranulation gene set, while the level of significance of pathway enrichment at each time point is indicated by color. Data are shown as median and interquartile range.

transcriptional signature of neutrophils transmigrated to CFASN, but not those transmigrated to LTB4. Among those, genes of the glycolytic and mTOR pathways and genes previously identified in blood myeloid-derived suppressor cells in cancer patients[28] were highly enriched in the former (Figure 2C). Moreover, of the gene subsets that were significantly different between CFASN and blood neutrophils (Figure S1C), 769 genes were significantly downregulated and 1,735 were significantly upregulated in CFASN compared to the LTB4 condition (Figure 2D). Interestingly, untargeted pathway enrichment analysis using the hallmarks (H), reactome (R), and Kyoto Encyclopedia of Genes and Genomes (KEGG) (K) datasets showed a clear downregulation of transcripts implicated in neutrophil and innate immune responses (Figure 2D) and a concomitant upregulation of metabolic pathways in CFASN-transmigrated neutrophils. This highlights the unique properties of that pathological milieu, which is necessary for the acquisition of the GRIM fate.

To validate these transcriptional findings, we sought to determine whether they were mirrored by proteomic changes. Proteomic results of blood neutrophils (pre-transmigration), and both CFASN- and LTB4-transmigrated neutrophils showed a core of 1,798 common proteins, some of which were shared only between 2 conditions, and a pool of proteins unique to each condition (Figures S3D and S3E). Pathway enrichment analysis of the unique proteins revealed overlap with transcriptional signatures, with protein enriched for antimicrobial and cell death pathways in blood neutrophils, while in CFASN-transmigrated neutrophils, proteins related to the transcriptional activation of immunomodulatory and anabolic pathways were enriched. These findings support the hypothesis that neutrophil conditioning after recruitment into the CF airway microenvironment and subsequent acquisition of the GRIM fate are driven by *de novo* transcription and translation.

Acquisition of the GRIM fate by CF airway neutrophils occurs in stepwise fashion

Since most transcriptional and functional changes seen in CF airway neutrophils occur over the course of several hours, we performed a kinetic assay to better understand the dynamics of the acquisition of the GRIM fate. Using the *in vitro* model, airway neutrophils were collected after 1, 2, 4, and 6 h of transmigration toward CFASN. We observed that the GRIM fate developed along a rapid time course, with stepwise increases in the release of primary granules (Figure 3A), loss of the phagocytic receptor CD16 (data not shown), and increase in the total RNA content compared to blood (Figure 3B) and LTB4 (Figure S4A), suggesting a co-regulation of degranulation and transcription. Analysis of the transcriptome of CFASN-transmigrated compared to blood neutrophils showed profound changes as early as 1 and 2 h post-transmigration (Figure 3C), with the upregulation of specific genes occurring before the significant downregulation of other gene modules. Further analysis revealed an upregulation over time of genes related to transcription, translation, and metabolism (Figure S4B). Interestingly,

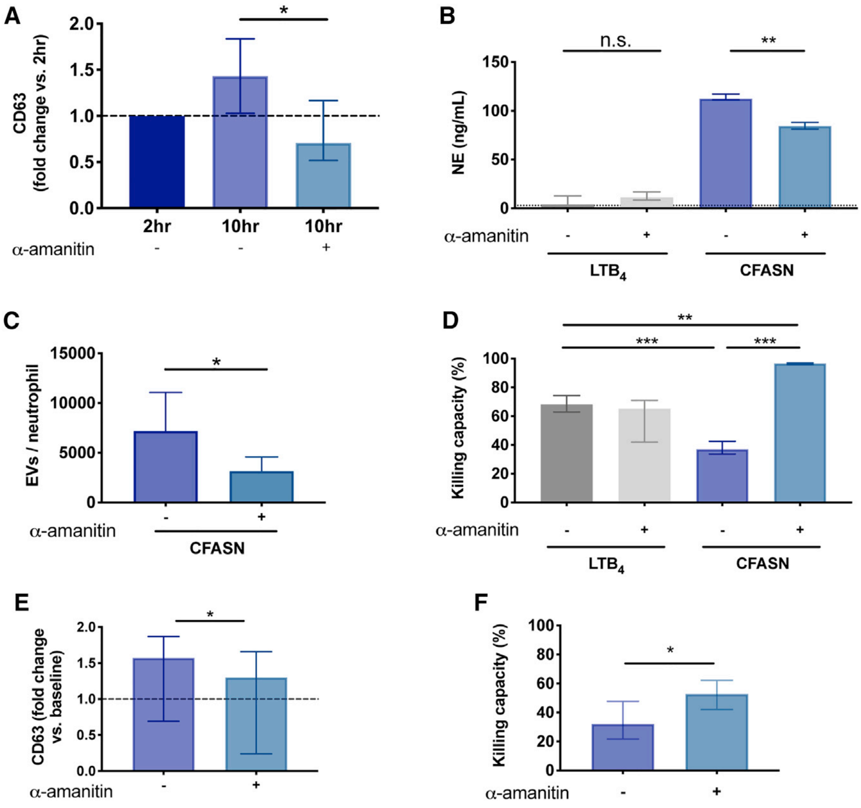

Figure 4. Functional adaptation to the CF airway microenvironment depends upon *de novo* transcription

Blood neutrophils (red) were transmigrated *in vitro* for 2 h toward CFASN and subsequently incubated for 8 h with or without the transcriptional blocker α-amanitin (N = 3 independent experiments).

(A–D) CFASN transmigrated neutrophils treated with α-amanitin showed reduced release of primary granules (A), concomitant with lower presence of NE in the extracellular milieu (B), reduced secretion of extracellular vesicles (EVs) (C), and increased killing of *Pseudomonas aeruginosa* (D).

(E and F) Short-term treatment of primary CF sputum neutrophils with α-amanitin (N = 3 independent experiments) led to similar patterns of reduced exocytosis (E) and increased killing of *P. aeruginosa* (F).

Data are shown as median and interquartile range and analyzed using the Wilcoxon matched-pairs signed rank test; *$p < 0.05$, **$p < 0.01$, ***$p < 0.001$.

among the pathways that were significantly downregulated compared to blood (Figure S4C; Table S3), genes belonging to the neutrophil degranulation reactome gene set were prominent (Figure 3D), mirroring the difference previously observed between CFASN and LTB4 neutrophils (Figure 2D). Of those genes, the majority of downregulated genes coded for effector proteins required for host defense against foreign microorganisms (Figure 3D), suggesting a possible relationship between transcription and neutrophil ability to clear pathogens.

Transcriptional blockade antagonizes the GRIM fate and restores bactericidal activity in CF airway neutrophils

To investigate a potential causal relationship between *de novo* transcription and GRIM fate acquisition, we transmigrated neutrophils in the model for 2 h into CFASN and then treated them with the RNA polymerase II and III-specific transcriptional blocker α-amanitin (Figure S5A). As expected, the total RNA content was reduced upon α-amanitin treatment (Figure S5B), showing the effectiveness of the blockade. Analysis of the transcriptome of neutrophils treated with α-amanitin revealed the downregulation of transcriptional and translational pathways, consistent with the effectiveness of the treatment. Furthermore, blockade of transcription by α-amanitin led to the upregulation of neutrophil degranulation and antibacterial genes compared to non-treated neutrophils (Figure S5C). Among the former, treatment with α-amanitin led to the upregulation of 20% of those genes that were significantly downregulated at 6 h post-transmigration. Of the genes recovered upon α-amanitin treatment, the majority belonged to proteins contained in the secretory vesicles (49%), followed by secondary granules (20%) and ficolin-rich granules (19%), suggesting that the blockade of *de novo* transcription may modulate the functional adaptation of neutrophils in CF airways.

To confirm that α-amanitin-induced changes in transcriptional profile were reflected in the functional changes of neutrophils in the CF airway microenvironment, we turned our attention to phenotypic analysis. Interestingly, α-amanitin induced a reduction in surface CD63 expression (Figure 4A), suggesting a decreased release of primary granules, which was confirmed by the quantification of NE in the extracellular milieu (Figure 4B). Moreover, a reduction in the release of extracellular vesicles (EVs), which were previously shown to promote tissue damage[29] was also observed upon transcriptional blockade by α-amanitin (Figure 4C), suggesting a relationship between transcriptional regulation and both granule and EV release.

Finally, since CFASN-transmigrated, α-amanitin-treated neutrophils showed decreased exocytosis and a concomitant upregulation of effector granule proteins, we questioned whether this would affect their bactericidal activity. To this end, we incubated α-amanitin-treated and -untreated neutrophils with *Pseudomonas aeruginosa*, a common CF pathogen, and assessed total bacterial count in an *in vitro* co-culture assay after 30 min. Remarkably, the bactericidal activity of CFASN-transmigrated neutrophils increased from 40% to 95% with α-amanitin treatment. Meanwhile, LTB4-transmigrated neutrophils remained stable at 65% bactericidal activity whether treated or not with α-amanitin, suggesting that the effects of α-amanitin are confined to CFASN-transmigrated neutrophils and do not perturb bacterial growth per se (Figure 4D).

To provide *in vivo* validation for the reduced exocytosis and gain of bactericidal activity observed with α-amanitin treatment *in vitro*, sputum neutrophils collected from patients with stable CF disease were treated with α-amanitin in a short-term culture

(2 h), followed by flow cytometry analysis or incubation with *P. aeruginosa* (30 min). Consistent with our *in vitro* findings, the blockade of *de novo* transcription by α-amanitin reduced neutrophil exocytosis (Figure 4E) and increased bactericidal activity by primary CF airway neutrophils from 32% to 53% (Figure 4F). These results suggest that *de novo* transcription and translation are key to driving the neutrophil adaptation (GRIM fate) observed in the CF airway milieu, in part via active repression of their bactericidal activity.

DISCUSSION

Neutrophil adaptation to the CF airway microenvironment features profound changes at the metabolic and functional levels, including increased numbers of polyribosomes, increased glycolytic activity, activation of the mTOR pathway, active release of primary granules, immune modulation, and decreased bacterial killing.[17–20] Our findings here demonstrate that this adaptation is driven by active transcription and translation and highlight the plasticity of neutrophils at the target organ.

Our findings provide an explanation for paradoxical bacterial infections occurring in the face of neutrophil recruitment and sustained metabolism of these cells in inflammatory sites such as the CF lung. This conundrum has traditionally been approached from the ability of the microorganisms to manipulate and escape the immune system. Here, we show that the local airway microenvironment is able to condition recruited neutrophils by inducing quick and extensive *de novo* transcription and translation.

Moreover, we show that blocking *de novo* transcription in airway neutrophils produced in an organotypic *in vitro* model and in short-term *ex vivo* culture of patients' airway neutrophils, restores their bactericidal activity, unveiling a surprising mechanism by which this canonical function of neutrophils is modulated. Combined with our prior discovery that neutrophilic inflammation occurs before chronic infection in CF infants,[16] these findings suggest a cascade of events in which infection may be facilitated by preexisting adaptations of tissue neutrophils. As evidenced by the decrease in expression of genes related to innate immune and cytokine signaling, we speculate that the cells may be actively conditioned to respond to types of stressors other than bacteria. This behavior, persisting into later stages, would contribute to the permissivity of CF airways to bacteria.[30]

Neutrophil plasticity at the site of inflammation is becoming increasingly apparent. In addition to known transcriptional changes in the bone marrow, there is a growing body of literature describing *de novo* transcription of inflammatory genes upon neutrophil priming,[10,31] transcriptional firing during NETosis,[32,33] and in a variety of chronic diseases,[34–37] in which phenotypic and functional changes have been related to disease outcomes and therapeutic success. In line with our results, a prior study described the dynamic regulation of the neutrophil transcriptome during the transition from bone marrow to blood and upon migration to the site of inflammation in a mouse model,[38] further supporting the plastic nature of neutrophils and the impact of the local microenvironment on their transcriptional profile. However, while there is increasing evidence of neutrophil plasticity through the transcription of new genes, its impact on neutrophil phenotype and function remains poorly understood, particularly in human diseases.

Here, we showed that the bactericidal activity of neutrophils can be repressed through the expression of new genes, suggesting a more profound role of *de novo* transcription that reaches beyond the expression of defined inflammatory mediators. This observation introduces another concept in neutrophil biology, which to date has been widely underappreciated, in which neutrophil antimicrobial activity is not only dictated by extrinsic factors derived from local damage or pathogen-associated molecular patterns but also modulated intrinsically through active transcriptional regulation, thereby instructing an exclusive fate of increased metabolic activity and adaptation with lower bactericidal performance (GRIM fate). The identification of the factors leading to transcriptional firing and downstream GRIM adaptation is a major focus of ongoing investigations in our laboratory, which due to the richness and variety of factors present in CFASN are outside of the range of the present study. However, preliminary fractionation of CFASN with molecular weight cutoff filters suggest that components larger than 300 kDa are responsible for inducing the GRIM fate (data not shown). In all cases, transmigration is required for the induction of the GRIM fate, as direct incubation does not suffice to induce a major transcriptional burst in neutrophils. In a related study, we found that transmigration (but not incubation) of blood monocytes in CF airway fluid inhibits their bactericidal activity,[39] suggesting that this effect is not restricted to neutrophils. Another line of investigation in our laboratory relates to the potential effects of oxygen tension on the acquisition of GRIM fate by CF airway neutrophils. CF airways can develop pockets of profound hypoxia in which neutrophils are found to be enriched.[40,41] While GRIM neutrophils may consume oxygen to generate oxidants, they may not depend on it for metabolism, as they are competent for glycolysis,[18] for which oxygen is not required.

GRIM neutrophils displayed a transcriptional burst across distinct regions on all chromosomes, emphasizing the extensive nature to which these cells can remodel their gene expression profile within just a few hours. This happened, however, with the notable exception of NE and other granule effector proteins, which are produced in pre-mitotic bone marrow neutrophil precursors,[42] but not in GRIM neutrophils. This observed lack of *de novo* transcription of granule proteins is in concordance with previously published studies showing the absence of transcription factors key to granule biogenesis and of corresponding mRNAs in mature neutrophils.[43–45] The absence of specific transcripts encoding for granule effector proteins normally expressed at early stages of bone marrow development supports the notion that GRIM adaptation of CF airway neutrophils is not linked to overall chromatin derepression, but rather to tightly orchestrated reopening of certain chromatin domains. Focused follow-up studies of neutrophil epigenetics upon recruitment to target organs are needed to shed light on this question. Of particular importance is the identification of factors enabling such extensive and yet fast transcriptional derepression of the hypercondensed chromatin characteristic of blood neutrophils as that observed in our study.

In conclusion, our findings demonstrate the ability of human neutrophils to actively adapt their canonical functions upon cues from the local microenvironment through the expression of new genes, challenging the age-old paradigm that holds these cells as terminally differentiated, and with little opportunity for adaptation at the site of inflammation. Importantly, these transcriptional changes have a notable impact on neutrophil function in the inflamed airway, including their bactericidal activity, which is traditionally thought as a canonical role of neutrophils. Future studies investigating the time-dependent switch between heterochromatin and euchromatin states will provide new insights in basic neutrophil biology and may open opportunities for host-directed immunotherapies modulating neutrophil behavior, rather than seeking to completely abrogate the benefits of neutrophil presence at a site of infection in CF and similar conditions.

Limitations of study

There are several limitations to this study. First, due to coronavirus disease 2019 (COVID-19) restrictions related to in-person visits for patients with CF, the sample size for validation experiments was suboptimal. Second, while our study mounts further evidence for an anabolic activation of neutrophils upon recruitment to the CF airway microenvironment, it does not formally establish whether the survival of these cells is increased *in vivo*. Isotope pulse/chase combined with mathematical modeling is needed to directly measure CF blood and airway neutrophil turnover/lifespan *in vivo*, and this question remains unresolved until such an effort is initiated. Third, our study did not test the potential impact of blood neutrophil heterogeneity on their behavior post-transmigration. Our prior work[18] and the present study established that the origin of blood neutrophils (from healthy or CF donors) does not affect the acquisition of the GRIM fate *in vitro*. Instead, CF ASN is the dominant factor in the acquisition of the GRIM fate, mirroring the dominant effect on epithelial interleukin-8 (IL-8) secretion exerted by CF mucopurulent medium on healthy control epithelial cultures in prior work by Ribeiro and colleagues.[46] Nevertheless, it remains to be determined whether discrete neutrophil subsets that may arise in the blood of patients with CF (e.g., band cells present during acute exacerbations) would respond differently in our model. Fourth, our prior work[18,47] and the findings of the present study suggest that GRIM adaptation of lung-recruited neutrophils is prominent in CF but may also occur in other chronic airway diseases. However, in-depth studies are required to fully discern shared and unique features conferred to lung-recruited neutrophils in distinct pathological microenvironments. This is a key question, since GRIM neutrophils are likely playing important protective functions, such as the inhibition of potential T cell-driven autoimmunity, notably via the induction of myeloid-derived suppressor cell (MDSC)-like responses, as evidenced here, including the release of arginase-I.[17] Therefore, potential therapeutic interventions directed at these cells in CF and other diseases would have to be carefully calibrated to recover defective bacterial killing and limit the effects of extracellular elastase and MPO, while preserving the beneficial functions of these tissue-recruited neutrophils.

STAR★METHODS

Detailed methods are provided in the online version of this paper and include the following:

- KEY RESOURCES TABLE
- RESOURCE AVAILABILITY
  - Lead contact
  - Materials availability
  - Data and code availability
- EXPERIMENTAL MODEL AND SUBJECT DETAILS
  - Human subjects and sample collection
  - H441 cell line
- METHOD DETAILS
  - *In vitro* transmigration
  - *Ex vivo* transcriptional blockade in CF airway neutrophils
  - Neutrophil elastase activity
  - Nanoparticle tracking analysis of extracellular vesicles
  - Flow cytometry
  - Microarrays
  - RNA sequencing
  - Fluidigm array
  - Proteomics
  - Bacterial killing
- QUANTIFICATION AND STATISTICAL ANALYSIS
  - Statistical analysis

SUPPLEMENTAL INFORMATION

Supplemental information can be found online at https://doi.org/10.1016/j.xcrm.2021.100239.

ACKNOWLEDGMENTS

This study was supported by a pilot grant from the Center for Pediatric Innovation at Georgia Tech & Emory to R.T. and G.G., CFF to R.T. (TIROUV15A0 and TIROUV19G0), CF@LANTA RDP Fellowship funded under MCCART15A0 to C.M., CF UAB RDP Fellowship funded under ROWE19R0 to C.M., and NIH/NHLBI to R.T. (R01HL126603). We thank the genomics core at the Yerkes Primate Research Center (Emory University) and the Emory University Pediatrics Department and Winship Cancer Institute flow cytometry core for their cooperation. CF human subject samples were provided by the CF Biospecimen Repository at the Children's Healthcare of Atlanta and Emory University CF Discovery Core.

AUTHOR CONTRIBUTIONS

C.M. and R.T. conceived the study; C.M., D.A.G., B.D., V.D.G., O.A.F., and F.S. collected the data; C.M., B.D., D.M.-G., H.K., and C.G. analyzed the data; A.G., R.W., and G.G. contributed the methods; C.M. and R.T. drafted the manuscript; and all of the authors reviewed and approved the final submission.

DECLARATION OF INTERESTS

The authors declare no competing interests.

INCLUSION AND DIVERSITY

We worked to ensure gender balance in the recruitment of human subjects. One or more of the authors of this paper self-identifies as an underrepresented ethnic minority in science. The author list of this paper includes contributors

from the location where the research was conducted who participated in the data collection, design, analysis, and/or interpretation of the work.

Received: January 23, 2020
Revised: November 15, 2020
Accepted: March 15, 2021
Published: April 8, 2021

REFERENCES

1. Lawrence, S.M., Corriden, R., and Nizet, V. (2018). The ontogeny of a neutrophil: mechanisms of granulopoiesis and homeostasis. Microbiol. Mol. Biol. Rev. *82*, e00057-17.
2. Summers, C., Rankin, S.M., Condliffe, A.M., Singh, N., Peters, A.M., and Chilvers, E.R. (2010). Neutrophil kinetics in health and disease. Trends Immunol. *31*, 318–324.
3. Paul, F., Arkin, Y., Giladi, A., Jaitin, D.A., Kenigsberg, E., Keren-Shaul, H., Winter, D., Lara-Astiaso, D., Gury, M., Weiner, A., et al. (2016). Transcriptional heterogeneity and lineage commitment in myeloid progenitors. Cell *164*, 325.
4. Borregaard, N. (2010). Neutrophils, from marrow to microbes. Immunity *33*, 657–670.
5. Taschner, S., Koesters, C., Platzer, B., Jörgl, A., Ellmeier, W., Benesch, T., and Strobl, H. (2007). Down-regulation of RXRalpha expression is essential for neutrophil development from granulocyte/monocyte progenitors. Blood *109*, 971–979.
6. Bartels, M., Govers, A.M., Fleskens, V., Lourenço, A.R., Pals, C.E., Vervoort, S.J., van Gent, R., Brenkman, A.B., Bierings, M.B., Ackerman, S.J., et al. (2015). Acetylation of C/EBPε is a prerequisite for terminal neutrophil differentiation. Blood *125*, 1782–1792.
7. Downey, G.P., Fukushima, T., Fialkow, L., and Waddell, T.K. (1995). Intracellular signaling in neutrophil priming and activation. Semin. Cell Biol. *6*, 345–356.
8. Miralda, I., Uriarte, S.M., and McLeish, K.R. (2017). Multiple phenotypic changes define neutrophil priming. Front. Cell. Infect. Microbiol. *7*, 217.
9. Jacobson, E.C., Perry, J.K., Long, D.S., Olins, A.L., Olins, D.E., Wright, B.E., Vickers, M.H., and O'Sullivan, J.M. (2018). Migration through a small pore disrupts inactive chromatin organization in neutrophil-like cells. BMC Biol. *16*, 142.
10. Wright, H.L., Thomas, H.B., Moots, R.J., and Edwards, S.W. (2013). RNA-seq reveals activation of both common and cytokine-specific pathways following neutrophil priming. PLoS ONE *8*, e58598.
11. Dwyer-Lindgren, L., Bertozzi-Villa, A., Stubbs, R.W., Morozoff, C., Shirude, S., Naghavi, M., Mokdad, A.H., and Murray, C.J.L. (2017). Trends and patterns of differences in chronic respiratory disease mortality among US counties, 1980-2014. JAMA *318*, 1136–1149.
12. Lozano, R., Naghavi, M., Foreman, K., Lim, S., Shibuya, K., Aboyans, V., Abraham, J., Adair, T., Aggarwal, R., Ahn, S.Y., et al. (2012). Global and regional mortality from 235 causes of death for 20 age groups in 1990 and 2010: a systematic analysis for the Global Burden of Disease Study 2010. Lancet *380*, 2095–2128.
13. Tirouvanziam, R. (2006). Neutrophilic inflammation as a major determinant in the progression of cystic fibrosis. Drug News Perspect. *19*, 609–614.
14. Tirouvanziam, R., Gernez, Y., Conrad, C.K., Moss, R.B., Schrijver, I., Dunn, C.E., Davies, Z.A., Herzenberg, L.A., and Herzenberg, L.A. (2008). Profound functional and signaling changes in viable inflammatory neutrophils homing to cystic fibrosis airways. Proc. Natl. Acad. Sci. USA *105*, 4335–4339.
15. Tirouvanziam, R., Khazaal, I., and Péault, B. (2002). Primary inflammation in human cystic fibrosis small airways. Am. J. Physiol. Lung Cell. Mol. Physiol. *283*, L445–L451.
16. Margaroli, C., Garratt, L.W., Horati, H., Dittrich, A.S., Rosenow, T., Montgomery, S.T., Frey, D.L., Brown, M.R., Schultz, C., Guglani, L., et al. (2019). Elastase exocytosis by airway neutrophils is associated with early lung damage in children with cystic fibrosis. Am. J. Respir. Crit. Care Med. *199*, 873–881.
17. Ingersoll, S.A., Laval, J., Forrest, O.A., Preininger, M., Brown, M.R., Arafat, D., Gibson, G., Tangpricha, V., and Tirouvanziam, R. (2015). Mature cystic fibrosis airway neutrophils suppress T cell function: evidence for a role of arginase 1 but not programmed death-ligand 1. J. Immunol. *194*, 5520–5528.
18. Forrest, O.A., Ingersoll, S.A., Preininger, M.K., Laval, J., Limoli, D.H., Brown, M.R., Lee, F.E., Bedi, B., Sadikot, R.T., Goldberg, J.B., et al. (2018). Frontline Science: pathological conditioning of human neutrophils recruited to the airway milieu in cystic fibrosis. J. Leukoc. Biol. *104*, 665–675.
19. Makam, M., Diaz, D., Laval, J., Gernez, Y., Conrad, C.K., Dunn, C.E., Davies, Z.A., Moss, R.B., Herzenberg, L.A., Herzenberg, L.A., and Tirouvanziam, R. (2009). Activation of critical, host-induced, metabolic and stress pathways marks neutrophil entry into cystic fibrosis lungs. Proc. Natl. Acad. Sci. USA *106*, 5779–5783.
20. Laval, J., Touhami, J., Herzenberg, L.A., Conrad, C., Taylor, N., Battini, J.L., Sitbon, M., and Tirouvanziam, R. (2013). Metabolic adaptation of neutrophils in cystic fibrosis airways involves distinct shifts in nutrient transporter expression. J. Immunol. *190*, 6043–6050.
21. Valvezan, A.J., Turner, M., Belaid, A., Lam, H.C., Miller, S.K., McNamara, M.C., Baglini, C., Housden, B.E., Perrimon, N., Kwiatkowski, D.J., et al. (2017). mTORC1 couples nucleotide synthesis to nucleotide demand resulting in a targetable metabolic vulnerability. Cancer Cell *32*, 624–638.e5.
22. Albert, V., and Hall, M.N. (2015). mTOR signaling in cellular and organismal energetics. Curr. Opin. Cell Biol. *33*, 55–66.
23. Aramburu, J., Ortells, M.C., Tejedor, S., Buxadé, M., and López-Rodríguez, C. (2014). Transcriptional regulation of the stress response by mTOR. Sci. Signal. *7*, re2.
24. Chandler, J.D., Margaroli, C., Horati, H., Kilgore, M.B., Veltman, M., Liu, H.K., Taurone, A.J., Peng, L., Guglani, L., Uppal, K., et al. (2018). Myeloperoxidase oxidation of methionine associates with early cystic fibrosis lung disease. Eur. Respir. J. *52*, 1801118.
25. Sagel, S.D., Kapsner, R.K., and Osberg, I. (2005). Induced sputum matrix metalloproteinase-9 correlates with lung function and airway inflammation in children with cystic fibrosis. Pediatr. Pulmonol. *39*, 224–232.
26. Sagel, S.D., Sontag, M.K., Wagener, J.S., Kapsner, R.K., Osberg, I., and Accurso, F.J. (2002). Induced sputum inflammatory measures correlate with lung function in children with cystic fibrosis. J. Pediatr. *141*, 811–817.
27. Rørvig, S., Østergaard, O., Heegaard, N.H., and Borregaard, N. (2013). Proteome profiling of human neutrophil granule subsets, secretory vesicles, and cell membrane: correlation with transcriptome profiling of neutrophil precursors. J. Leukoc. Biol. *94*, 711–721.
28. Condamine, T., Dominguez, G.A., Youn, J.I., Kossenkov, A.V., Mony, S., Alicea-Torres, K., Tcyganov, E., Hashimoto, A., Nefedova, Y., Lin, C., et al. (2016). Lectin-type oxidized LDL receptor-1 distinguishes population of human polymorphonuclear myeloid-derived suppressor cells in cancer patients. Sci. Immunol. *1*, aaf8943.
29. Genschmer, K.R., Russell, D.W., Lal, C., Szul, T., Bratcher, P.E., Noerager, B.D., Abdul Roda, M., Xu, X., Rezonzew, G., Viera, L., et al. (2019). Activated PMN exosomes: pathogenic entities causing matrix destruction and disease in the lung. Cell *176*, 113–126.e15.
30. Margaroli, C., and Tirouvanziam, R. (2016). Neutrophil plasticity enables the development of pathological microenvironments: implications for cystic fibrosis airway disease. Mol. Cell Pediatr. *3*, 38.
31. Zhang, X., Kluger, Y., Nakayama, Y., Poddar, R., Whitney, C., DeTora, A., Weissman, S.M., and Newburger, P.E. (2004). Gene expression in mature neutrophils: early responses to inflammatory stimuli. J. Leukoc. Biol. *75*, 358–372.
32. Khan, M.A., and Palaniyar, N. (2017). Transcriptional firing helps to drive NETosis. Sci. Rep. *7*, 41749.

33. Agraz-Cibrian, J.M., Giraldo, D.M., and Urcuqui-Inchima, S. (2019). 1,25-Dihydroxyvitamin D_3 induces formation of neutrophil extracellular trap-like structures and modulates the transcription of genes whose products are neutrophil extracellular trap-associated proteins: A pilot study. Steroids *141*, 14–22.

34. Tutino, V.M., Poppenberg, K.E., Jiang, K., Jarvis, J.N., Sun, Y., Sonig, A., Siddiqui, A.H., Snyder, K.V., Levy, E.I., Kolega, J., and Meng, H. (2018). Circulating neutrophil transcriptome may reveal intracranial aneurysm signature. PLoS ONE *13*, e0191407.

35. Naranbhai, V., Fairfax, B.P., Makino, S., Humburg, P., Wong, D., Ng, E., Hill, A.V., and Knight, J.C. (2015). Genomic modulators of gene expression in human neutrophils. Nat. Commun. *6*, 7545.

36. Kenyon, A., Gavriouchkina, D., Zorman, J., Napolitani, G., Cerundolo, V., and Sauka-Spengler, T. (2017). Active nuclear transcriptome analysis reveals inflammasome-dependent mechanism for early neutrophil response to Mycobacterium marinum. Sci. Rep. *7*, 6505.

37. Jiang, K., Sun, X., Chen, Y., Shen, Y., and Jarvis, J.N. (2015). RNA sequencing from human neutrophils reveals distinct transcriptional differences associated with chronic inflammatory states. BMC Med. Genomics *8*, 55.

38. Lakschevitz, F.S., Visser, M.B., Sun, C., and Glogauer, M. (2015). Neutrophil transcriptional profile changes during transit from bone marrow to sites of inflammation. Cell. Mol. Immunol. *12*, 53–65.

39. Ford, B.D., Moncada Giraldo, D., Margaroli, C., Giacalone, V.D., Brown, M.R., Peng, L., and Tirouvanziam, R. (2021). Functional and transcriptional adaptations of blood monocytes recruited to the cystic fibrosis airway microenvironment in vitro. Int. J. Mol. Sci. *22*, 2530.

40. Kolpen, M., Hansen, C.R., Bjarnsholt, T., Moser, C., Christensen, L.D., van Gennip, M., Ciofu, O., Mandsberg, L., Kharazmi, A., Döring, G., et al. (2010). Polymorphonuclear leucocytes consume oxygen in sputum from chronic Pseudomonas aeruginosa pneumonia in cystic fibrosis. Thorax *65*, 57–62.

41. Worlitzsch, D., Tarran, R., Ulrich, M., Schwab, U., Cekici, A., Meyer, K.C., Birrer, P., Bellon, G., Berger, J., Weiss, T., et al. (2002). Effects of reduced mucus oxygen concentration in airway *Pseudomonas* infections of cystic fibrosis patients. J. Clin. Invest. *109*, 317–325.

42. Grassi, L., Pourfarzad, F., Ullrich, S., Merkel, A., Were, F., Carrillo-de-Santa-Pau, E., Yi, G., Hiemstra, I.H., Tool, A.T.J., Mul, E., et al. (2018). Dynamics of transcription regulation in human bone marrow myeloid differentiation to mature blood neutrophils. Cell Rep. *24*, 2784–2794.

43. Fouret, P., du Bois, R.M., Bernaudin, J.F., Takahashi, H., Ferrans, V.J., and Crystal, R.G. (1989). Expression of the neutrophil elastase gene during human bone marrow cell differentiation. J. Exp. Med. *169*, 833–845.

44. Takahashi, H., Nukiwa, T., Basset, P., and Crystal, R.G. (1988). Myelomonocytic cell lineage expression of the neutrophil elastase gene. J. Biol. Chem. *263*, 2543–2547.

45. Bjerregaard, M.D., Jurlander, J., Klausen, P., Borregaard, N., and Cowland, J.B. (2003). The in vivo profile of transcription factors during neutrophil differentiation in human bone marrow. Blood *101*, 4322–4332.

46. Ribeiro, C.M., Paradiso, A.M., Schwab, U., Perez-Vilar, J., Jones, L., O'neal, W., and Boucher, R.C. (2005). Chronic airway infection/inflammation induces a Ca2+i-dependent hyperinflammatory response in human cystic fibrosis airway epithelia. J. Biol. Chem. *280*, 17798–17806.

47. Grunwell, J.R., Giacalone, V.D., Stephenson, S., Margaroli, C., Dobosh, B.S., Brown, M.R., Fitzpatrick, A.M., and Tirouvanziam, R. (2019). Neutrophil dysfunction in the airways of children with acute respiratory failure due to lower respiratory tract viral and bacterial coinfections. Sci. Rep. *9*, 2874.

48. Oh, H., Siano, B., and Diamond, S. (2008). Neutrophil isolation protocol. J. Vis. Exp. *17*, 745.

49. Tirouvanziam, R., Diaz, D., Gernez, Y., Laval, J., Crubezy, M., and Makam, M. (2011). An integrative approach for immune monitoring of human health and disease by advanced flow cytometry methods. In Advanced Optical Flow Cytometry: Methods and Disease Diagnoses, V.V. Tuchin, ed. (Wiley-VCH Verlag).

50. Bolstad, B.M., Irizarry, R.A., Astrand, M., and Speed, T.P. (2003). A comparison of normalization methods for high density oligonucleotide array data based on variance and bias. Bioinformatics *19*, 185–193.

51. Kim, D., Paggi, J.M., Park, C., Bennett, C., and Salzberg, S.L. (2019). Graph-based genome alignment and genotyping with HISAT2 and HISAT-genotype. Nat. Biotechnol. *37*, 907–915.

52. Love, M.I., Huber, W., and Anders, S. (2014). Moderated estimation of fold change and dispersion for RNA-seq data with DESeq2. Genome Biol. *15*, 550.

53. Ernst, J., and Bar-Joseph, Z. (2006). STEM: a tool for the analysis of short time series gene expression data. BMC Bioinformatics *7*, 191.

54. Ernst, J., Nau, G.J., and Bar-Joseph, Z. (2005). Clustering short time series gene expression data. Bioinformatics *21* (*Suppl 1*), i159–i168.

55. Eng, J.K., McCormack, A.L., and Yates, J.R. (1994). An approach to correlate tandem mass spectral data of peptides with amino acid sequences in a protein database. J. Am. Soc. Mass Spectrom. *5*, 976–989.

CellPress
OPEN ACCESS

STAR★METHODS

KEY RESOURCES TABLE

REAGENT or RESOURCE	SOURCE	IDENTIFIER
Antibodies		
CD45	Biolegend	Cat#368523, RRID: AB_2810854-
CD66b	Biolegend	Cat#305107, RRID: AB_314496-
CD16	Biolegend	Cat#302043, RRID: AB_11219184-
CD63	Biolegend	Cat# 353003, RRID: AB_10896786-
Zombie Aqua	Biolegend	Cat#423101
Bacterial and virus strains		
P. aeruginosa, PAO1 (Xen41)	Perkin Elmer	Cat#119229
Biological samples		
Patient blood and sputum	CF Biospecimen Repository - Children's Healthcare of Atlanta and Emory University CF Discovery Core	https://www.pedsresearch.org/research/cores/cf-discovery-core/overview/
Patient blood and sputum	NAC Phase IIB Trial -Stanford University	https://clinicaltrials.gov/ct2/show/NCT00809094
Chemicals, peptides, and recombinant proteins		
DMEM/F-12	Corning	Cat#15-090-CV
Glutamine	Sigma	Cat#G7513
FBS	Corning	Cat#35-011-CV
Penicillin/Streptomycin	Sigma	Cat#P4333
Trypsin	Hyclone	Cat#SH30042.01
Rat tail collagen type I	Sigma	Cat#C3867-1VL
Ultroser-G	Crescent Chemical Co	Cat#67042
RPMI 1640	Corning	Cat#10-040-CV
CXCL-8	Biolegend	Cat#574202
LPS	Sigma	Cat#L9143
C5a	R&D systems	Cat#2037-C5-025/CF
LTB4	Sigma	Cat#L0517
NEmo-1 probe	Sirius Fine Chemicals SiChem GmbH	Cat#SC-0200
SYTO RNASelect	Thermofisher	Cat#S32703
α-amanitin	Sigma	Cat#A2263
Critical commercial assays		
Clontech Nucleospin RNA	Takara Bio	Cat#740955
PolymorphPrep	Cosmo Bio USA	Cat#AXS-1114683
WT-Ovation Pico RNA Amplification System	NuGen	Cat#3302
TruSeq RNA Single Indexes Set B kit	Illumina	Cat#20020493
AmPure beads	Beckman	Cat#A63881
Deposited data		
Proteomics data	This paper	https://doi.org/10.17632/vkz52nv6nv.2
Microarrays	This paper	https://doi.org/10.17632/vkz52nv6nv.2
10hr RNASeq	This paper	https://www.ncbi.nlm.nih.gov/geo/query/acc.cgi?acc=GSE167069
10hr RNASeq with transcriptional blockade	This paper	https://www.ncbi.nlm.nih.gov/geo/query/acc.cgi?acc=GSE167066
RNASeq kinetic experiment	This paper	https://www.ncbi.nlm.nih.gov/geo/query/acc.cgi?acc=GSE165265

(*Continued on next page*)

Continued

REAGENT or RESOURCE	SOURCE	IDENTIFIER
Experimental models: Cell lines		
NCI-H441	ATCC	Cat# HTB-174
Software and algorithms		
JMP v13	SAS Institute	https://www.jmp.com/en_us/software.html
Prism v7	GraphPad	https://www.graphpad.com/scientific-software/prism/
FlowJo v9.9.5	BD biosciences	https://www.flowjo.com/
R v3.5.2	R project	https://www.r-project.org/
Microsoft Excel	Microsoft	https://www.microsoft.com/en-us/microsoft-365/p/excel/cfq7ttc0k7dx?activetab=pivot:overviewtab
Adobe Illustrator 2020	Adobe	https://www.adobe.com/products/illustrator.html
Robust Multichip Average express software v1.1.0	Bioconductor	https://rdrr.io/bioc/xps/man/rma.html
GE2-v5	Agilent	https://www.agilent.com
hisat2	GitHub	https://github.com/DaehwanKimLab/hisat2
Bowtie2	Johns Hopkins University	http://bowtie-bio.sourceforge.net/bowtie2/index.shtml
Samtools	Genome Research Limited	http://samtools.sourceforge.net/
featureCounts	Bioconductor	https://bioconductor.org/packages/release/bioc/html/Rsubread.html
DESeq2	Bioconductor	https://bioconductor.org/packages/release/bioc/html/DESeq2.html
Qlucore Omix Explorer v3.3	Qlucore	https://qlucore.com/
Short Time-series Expression Miner (STEM, v1.3.12)	Carnegie Mellon University, School of Computer Science	https://www.cs.cmu.edu/~jernst/stem/
GSEA	Broad Insititute	https://www.gsea-msigdb.org/gsea/index.jsp
SEQUEST algorithm	ThermoFisher	https://www.thermofisher.com/order/catalog/product/OPTON-30945?SID=srch-srp-OPTON-30945#/OPTON-30945?SID=srch-srp-OPTON-30945
Ideogram	GitHub	https://github.com/eweitz/ideogram
Other		
Alvetex scaffold for *in vitro* model	Reprocell	Cat#AVP005

RESOURCE AVAILABILITY

Lead contact

Further information and requests for resources and reagents should be directed to and will be fulfilled by the Lead Contact, Dr. Rabindra Tirouvanziam (tirouvanziam@emory.edu).

Materials availability

This study did not generate new unique reagents.

Data and code availability

The datasets generated during this study are available on Mendeley for microarrays and proteomics. https://doi.org/10.17632/vkz52nv6nv.2.

Original data for RNASeq analyses are available on GEO Accession viewer at GSE167069 (10 hours transmigration), GSE165265 (kinetics assay), GSE167066 (transcriptional blockade).

EXPERIMENTAL MODEL AND SUBJECT DETAILS

Human subjects and sample collection

Blood was obtained from healthy donors and age-matched patients with CF by venipuncture in K_2-EDTA tubes (demographics in Table S1). The study was approved by our Institutional Review Board. Airway neutrophils and sputum supernatant were obtained as previously published.[18] Briefly, expectorated sputum from CF patients was solubilized with 2.5 mM final ice-cold PBS-EDTA and gently passaged through an 18.5 G needle. Airway neutrophils were recovered after an 800 g, 10 minutes, 4°C centrifugation, while the CFASN (clear supernatant) was collected following an additional 3,000 g, 10 minutes, 4°C centrifugation.

H441 cell line

H441 cells were purchased from ATCC (Cat# HTB-174) and cultured in DMEM/F-12 media supplemented with 10% FBS (Corning), 2 mM glutamine (Sigma) and 100 U/mL-0.1 mg/mL penicillin/streptomycin (Sigma). H441 cells from passages 8-10 were harvested and used for the *in vitro* transmigration model as previously detailed.[18] Briefly, 2.5 x10^5 H441 were cultured at air-liquid interphase on the Alvetex scaffold (Reprocell) coated with rat tail collagen (Sigma) for 14 days. The DMEM/F-12 media supplemented with 2% Ultroser G (Crescent Chemical Co), 2 mM glutamine (Sigma) and 100 U/mL-0.1mg/mL penicillin/streptomycin (Sigma), at the basolateral side was replaced every two days. After two weeks the membranes were inversed to allow the loading of neutrophils on the basolateral side and migration into apical fluid as described below.

METHOD DETAILS

In vitro transmigration

Blood neutrophils were isolated using the density gradient Polymorphprep (Cosmo Bio USA) following manufacturer protocol.[48] Neutrophil purity and viability was assessed using flow cytometry. Purified neutrophils were loaded in the *in vitro* transmigration model[18] as follows: 1) transmigration toward the neutrophil chemoattractant LTB4 (100nM, Sigma) (transmigrated neutrophils not undergoing GRIM adaptation); 2) transmigration using the CF airway supernatant (CFASN) generated from sputum as apical fluid, (transmigrated neutrophils undergoing GRIM adaptation); 3) transmigration using the COPD airway supernatant (COPDASN) generated from sputum as apical fluid; 4) CXCL-8 (Biolegend, 10 ng/mL); 5) LPS (Sigma, 10 ng/mL); and C5a (R&D systems, 500 ng/mL). Neutrophils were then collected at 1, 2, 4, 6 and 10 hours post-transmigration and used for downstream assays. Blockade of transcription was performed using neutrophils transmigrated for 2 hours, washed cells were incubated in RPMI for 8 hours with or without the transcriptional blocker α-amanitin (Sigma) at 1 μg/mL per 1 million cells. After incubation cells were assessed for their bacterial killing capacity and their phenotype determined by flow cytometry as described previously.[18]

Ex vivo transcriptional blockade in CF airway neutrophils

Neutrophils obtained from sputum as described above were incubated with the transcriptional blocker α-amanitin (Sigma) at 1 μg/mL per 1 million cells in RPMI for 2 hours at 37°C, 5% CO_2. Cells were washed and analyzed by flow cytometry or used for bacterial killing assays.

Neutrophil elastase activity

Free extracellular NE activity was measured using the FRET-based NEmo-1 probe (Sirius Fine Chemicals SiChem GmbH), as previously described.[16] Samples were measured in duplicate with repeated-measures for values outside the working range.

Nanoparticle tracking analysis of extracellular vesicles

Concentration and size of extracellular vesicles (EVs) was determined by Nanosight NS3000 (Malvern Panalytical). Size and median concentration are illustrated in Figure S5.

Flow cytometry

Cells were stained with surface markers and dyes as previously described[49]: CD63, CD66b, CD15, CD16, Live/dead zombie dye (Biolegend). Total RNA content was quantified by SytoRNA (ThermoFisher). Cells were acquired on a LSRII cytometer (BD Biosciences) and results were analyzed by FlowJo v.9.9.5 (BD Biosciences).

Microarrays

Blood and sputum neutrophils were sorted from patients with CF (N = 7; Table S1) as previously described.[20] Total RNA was extracted from sorted fractions, mRNA was amplified, and cDNA generated (WT-Ovation Pico RNA Amplification System, NuGen). Next, cDNA from sorted neutrophils from sputum and blood sample pairs was labeled with Cy3 and Cy5, respectively, to generate two-color microarray data by competitive hybridization (GE2-v5, Agilent), per manufacturer's instructions. RNA profiling data were

normalized using quantile-normalization with the Robust Multichip Average (RMA) express software v1.1.0,[50] and analyzed using Gene Set Enrichment Analysis (GSEA, Broad Institute) for significant enrichment of GO terms.

RNA sequencing

RNA was isolated from purified (blood > 98% purity) and transmigrated (LTB4 or CFASN, > 98% purity) neutrophils using the Nucleospin RNA isolation kit (Clontech, Takara Bio) following manufacturer protocol. RNA quality and concentration were quantified using a Bioanalyzer (Agilent Technologies, Inc.) and only samples with RIN > 8.0 were used for sequencing. Libraries were prepared using the TruSeq RNA Single Indexes Set B kit (Illumina) and AmPure beads (Beckman) and sequenced as paired end (100bp) by high seq rapid run (Illumina) with an aim of ~20 million reads per sample. Analysis of transcriptomics data was performed using hisat2 for the alignment to the reference genome GRCh39.[51] Differential gene expression between conditions was determined using DESeq2[52] and a significance was threshold was set at 0.01 for the adjusted p value (Benjamini and Hochberg adjustment for multiple testing), PCA was performed using the Qlucore Omix Explorer v3.3, the Short Time-series Expression Miner (STEM, v1.3.12) for the kinetic assay analysis,[53,54] and GSEA for pathway enrichment analysis (Broad Institute).

Fluidigm array

48 target genes, including NE, MPO, MMP9 and arginase-1, were assessed using a Fluidigm array, and multiplexed qPCR was performed on mRNA from blood and CF sputum neutrophils, as previously described.[17] mRNA levels in Figure S2B are shown as cycle quantification value (Cq), with 30 cycles being the lower detection limit.

Proteomics

Proteins were extracted from purified (blood > 98% purity) and transmigrated (LTB4 or CFASN, > 98% purity) neutrophils after cell lysis, followed by reduction and alkylation, and digestion with trypsin. The samples were separated into ten fractions using high-pH reversed phase high-performance liquid chromatography (HPLC, pH = 10). Each fraction was analyzed by an online liquid chromatography / tandem mass spectrometry (LC-MS/MS, LTQ Orbitrap Elite) system. The spectra were searched against a human proteome database using the SEQUEST algorithm.[55] Each protein sequence was listed in both forward and reversed orientations to estimate the false discovery rate (FDR) of peptide and protein identifications. The following parameters were used for the search: 10 ppm precursor mass tolerance; 0.5 Da product ion mass tolerance; fully digested with Lys-C; up to two missed cleavages; variable modifications: oxidation of methionine (+15.9949); fixed modifications: carbamidomethylation of cysteine (+57.0214). Peptides with fewer than seven amino acids were deleted, and peptide spectral matches were filtered to < 1% FDR. In addition, a protein-level filter was further performed in each dataset to reduce the protein-level FDR to be < 1%.

Bacterial killing

Overnight cultures of pro-inflammatory bacteria *P. aeruginosa* (strain PAO1, Perkin Elmer) were sub-aliquoted and grown to reach the exponential growth phase. Bacteria were then incubated in RPMI, supplemented with 10% FBS, on an end-over-end rotating wheel for 30 minutes at 37°C. Transmigrated neutrophils (10^5 cells) were resuspended in RPMI, 10% FBS and incubated at 37°C, 5% CO_2 for 15 minutes. Co-incubation of bacteria and leukocytes was performed at a multiplicity of infection of 0.1, in RPMI, 10% FBS, on an end-over-end rotating wheel for 30 minutes at 37°C, 5% CO_2. Bacterial killing capacity was calculated using colony forming units (CFU), with the bacteria plus RPMI and 10% FBS condition set as 100% viability.

QUANTIFICATION AND STATISTICAL ANALYSIS

Statistical analysis

Data were compiled in Excel (Microsoft) and transferred to JMP13 (SAS Institute) and Prism v7 (GraphPad) for statistical analysis and graphing, respectively. Differential gene expression between conditions was determined using DESeq2[52] in R v3.5.2 and a significance was threshold was set at 0.01 for the adjusted p value (Benjamini and Hochberg adjustment for multiple testing). Principal component analysis (PCA) was performed using the Qlucore Omix Explorer v3.3, the Short Time-series Expression Miner (STEM, v1.3.12) for the kinetic assay analysis,[53,54] and GSEA for pathway enrichment analysis (Broad Institute, FDR < 5%). For proteomics analyses, both peptide and protein FDRs thresholds were set at < 1%. Comparison between conditions were performed using non-parametric statistics (median, interquartile range). Statistical details can be found in the legend for each figure.

Cell Reports Medicine, Volume *2*

Supplemental information

Transcriptional firing represses bactericidal activity in cystic fibrosis airway neutrophils

Camilla Margaroli, Diego Moncada-Giraldo, Dalia Arafat Gulick, Brian Dobosh, Vincent D. Giacalone, Osric A. Forrest, Fangxu Sun, Chunhui Gu, Amit Gaggar, Haydn Kissick, Ronghu Wu, Greg Gibson, and Rabindra Tirouvanziam

Cell Reports Medicine – Research Article

TRANSCRIPTIONAL FIRING REPRESSES BACTERICIDAL ACTIVITY IN CYSTIC FIBROSIS AIRWAY NEUTROPHILS

Camilla Margaroli, Diego Moncada-Giraldo, Dalia Arafat Gulick,

Brian Dobosh, Vincent D. Giacalone, Osric A. Forrest, Fangxu Sun, Chunhui Gu,

Amit Gaggar, Haydn Kissick, Ronghu Wu, Greg Gibson, Rabindra Tirouvanziam

Supplemental information

Tables 3

Figures 5

CF patients<br>In vitro/RNASeq	Age	Gender	Ethnicity	CFTR genotype	Modulator Therapy
1	30	F	Caucasian	HO	Tezacaftor / Ivacaftor
2	24	M	Caucasian	HZ	None
3	37	M	Caucasian	HO	Tezacaftor / Ivacaftor
4	29	M	Caucasian	HO	None
5	22	F	Caucasian	OT	Tezacaftor / Ivacaftor
6	31	F	Caucasian	HZ	None
7	32	F	Asian	OT	None
**CF patients Microarrays**					
1	50	M	Caucasian	HO	None
2	32	F	Caucasian	HZ	None
3	12	F	Caucasian	HO	None
4	25	M	Caucasian	HO	None
5	32	M	Caucasian	OT	None
6	38	F	Caucasian	HZ	None
7	32	M	Caucasian	HO	None
**COPD patients**					
1	56	F	Caucasian	N/A	None
2	55	M	Caucasian	N/A	None
3	45	F	Caucasian	N/A	None
**Healthy donors**					
1	27	F	Caucasian	N/A	None
2	24	M	Caucasian	N/A	None
3	25	F	Caucasian	N/A	None
4	23	M	Caucasian	N/A	None
5	40	M	Caucasian	N/A	None

Table S1. Patient demographics related to STAR Methods. Legend: F, female; HO, homozygous for F508Del mutation; HZ, heterozygous for one F508Del mutation and another CFTR mutation; M, male; N/A, not available; OT, other mutations than F508Del.

		1 hour		2 hours		4 hours		6 hours	
Gene set name	Genes in gene set (K)	Genes in overlap (K)	FDR q-value	Genes in overlap (K)	FDR q-value	Genes in overlap (K)	FDR q-value	Genes in overlap (K)	FDR q-value
Gene expression transcription (R)	1486	19	0.0076	161	6.01E-16	0	NA	101	4.58E-45
Generic transcription pathway (R)	1218	17	0.0076	142	1.55E-16	0	NA	83	1.68E-36
Interferon gamma response (H)	200	0	NA	52	2.80E-19	35	1.64E-14	35	3.58E-28
Cytokine signaling in immune system (R)	856	0	NA	114	3.37E-17	59	6.89E-07	102	1.00E-68
Allograft rejection (H)	200	0	NA	39	2.47E-10	21	9.33E-05	31	2.95E-23
Adaptive immune system (R)	811	0	NA	117	5.83E-20	66	9.52E-11	93	8.96E-61
Innate immune system (R)	1104	0	NA	138	1.74E-18	111	3.52E-26	157	1.82E-122
Interferon alpha response (H)	97	0	NA	28	1.66E-11	19	2.46E-08	0	NA
Interferon signaling (R)	202	0	NA	38	1.13E-09	25	6.28E-07	0	NA
Signaling by interleukins (R)	631	0	NA	78	4.96E-10	0	NA	73	3.34E-47
Hemostasis (R)	674	0	NA	0	NA	50	9.51E-07	65	6.49E-37
Neutrophil degranulation (R)	478	0	NA	0	NA	64	6.48E-21	92	7.61E-81
Immunoregulatory interactions between a lymphoid and a non-lymphoid cell (R)	186	0	NA	0	NA	23	1.87E-06	28	1.15E-20
Generation of 2nd messenger molecules (R)	37	0	NA	15	2.24E-08	0	NA	0	NA
Heme metabolism (H)	200	0	NA	35	3.76E-08	0	NA	0	NA
Intracellular signaling by 2nd messengers (R)	302	0	NA	47	3.97E-09	0	NA	0	NA
Toll like receptor cascades (R)	153	0	NA	30	3.77E-08	0	NA	0	NA
MAPK signaling pathway (K)	267	0	NA	45	8.03E-10	0	NA	0	NA
T cell receptor signaling pathway (K)	108	0	NA	32	1.36E-13	0	NA	0	NA
Metabolism of lipids (R)	738	0	NA	84	3.97E-09	0	NA	0	NA
MAPK pathway (R)	81	0	NA	22	1.47E-08	0	NA	0	NA

Vesicle mediated transport (R)	723	0	NA	80	3.56E-08	0	NA	0	NA
Chromatin organization (R)	272	0	NA	44	4.14E-09	0	NA	0	NA
Signaling by Rho GTPases (R)	450	0	NA	0	NA	33	2.10E-04	0	NA
Platelet homeostasis (R)	86	0	NA	0	NA	13	1.71E-04	0	NA
Beta catenin independent Wnt signaling (R)	145	0	NA	0	NA	17	1.71E-04	0	NA
Interferon alpha beta signaling (R)	70	0	NA	0	NA	12	1.25E-04	0	NA
Leishmania infection (K)	72	0	NA	0	NA	13	3.25E-05	0	NA
Fc gamma R mediated phagocytosis (K)	96	0	NA	0	NA	14	1.25E-04	0	NA
ESR mediated signaling (R)	219	0	NA	0	NA	23	3.25E-05	0	NA
Signaling by Notch (R)	244	0	NA	0	NA	23	1.51E-04	0	NA
Transport of small molecules (R)	728	0	NA	0	NA	48	4.62E-05	0	NA
Signaling by Wnt (R)	327	0	NA	0	NA	27	2.09E-04	0	NA
Complement (H)	200	0	NA	0	NA	0	NA	36	2.00E-29
Regulation of actin cytoskeleton (K)	213	0	NA	0	NA	0	NA	30	2.80E-21
Inflammatory response (H)	200	0	NA	0	NA	0	NA	31	2.95E-23
Signaling by receptor tyrosine kinases (R)	468	0	NA	0	NA	0	NA	48	3.94E-28
Chemokine signaling pathway (K)	189	0	NA	0	NA	0	NA	30	8.06E-23
TNFa signaling via NFkB (H)	200	0	NA	0	NA	0	NA	28	8.34E-20
Other interleukin signaling (R)	288	0	NA	0	NA	0	NA	32	1.24E-19
Class I MHC mediated antigen processing presentation (R)	370	0	NA	0	NA	0	NA	37	3.76E-21

Table S3. Kinetics of pathway downregulation in CFASN-migrated neutrophils (related to Figure 3 and Figure S6). The transcriptome of neutrophils was analyzed at 1, 2, 4, and 6 hours post-transmigration towards CFASN. Pathway enrichment analysis was conducted by GSEA on downregulated genes at each time point compared to blood (FDR q-value <5%). Legend: H, hallmarks; K, Kegg; R, reactome.

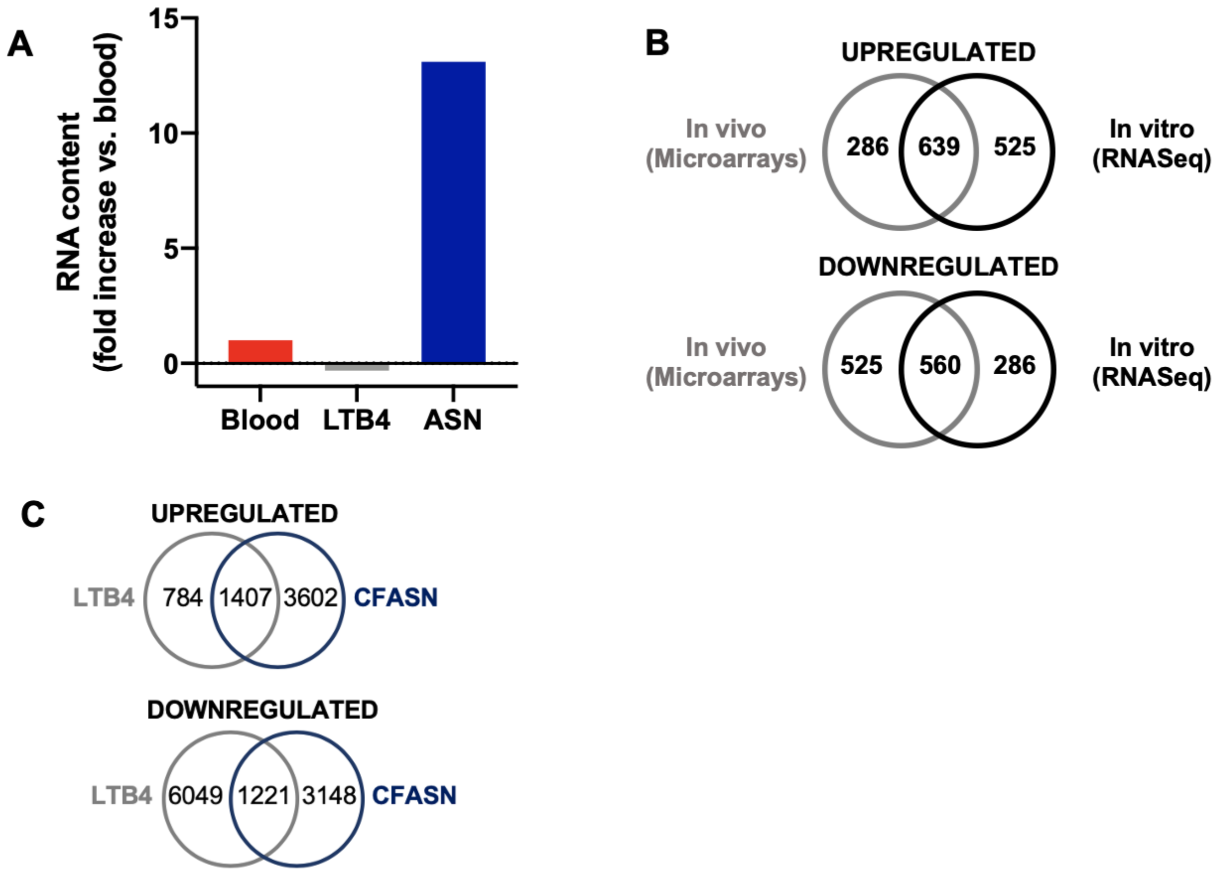

Figure S1 related to Figure 1. GRIM neutrophils show an increase in RNA content in the cystic fibrosis lung *in vivo* and *in vitro*. (A) Blood neutrophils from CF patients (red, N=1) were transmigrated (TM) *in vitro* using sputum supernatant (CFASN, blue) or the chemoattractant leukotriene B4 (LTB4) as a transmigration control (grey). Total RNA quantification was obtained using the bioanalyzer. **(B)** Genes that were commonly upregulated or downregulated both *in vivo* (microarrays) and *in vitro* (RNASeq) compared to blood. **(C)** Differential gene expression of upregulated and downregulated genes was determined comparing transmigrated conditions to blood. Gene expression regulation was determined by log2 gene expression greater than 2 and smaller than -2.

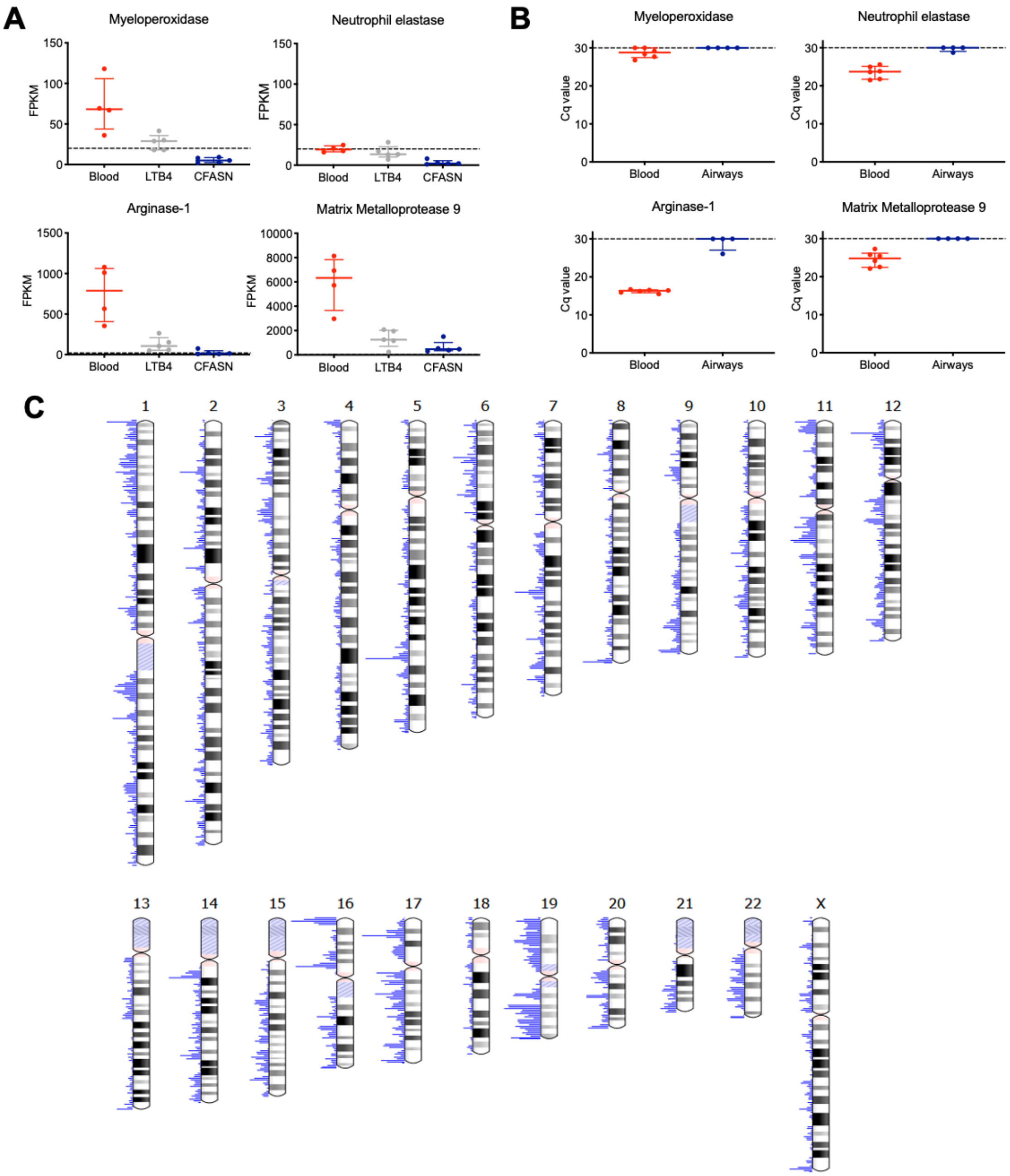

Figure S2 related to figure 2. CF airway neutrophils lack *de novo* expression of main granule effector proteins. RNA of effector granule proteins measured by RNASeq *in vitro* **(A)** and by Fluidigm *in vivo* **(B)** are not detectable in CF airway GRIM neutrophils. Dotted line represents lower limit of detection (20 FPKM for RNASeq, and 30 Ct for Fluidigm). **(C)** Histograms represent RNA counts of transcripts detected by RNASeq in neutrophils transmigrated to CFASN at their respective chromosomal positions showing the breadth of transcriptional firing.

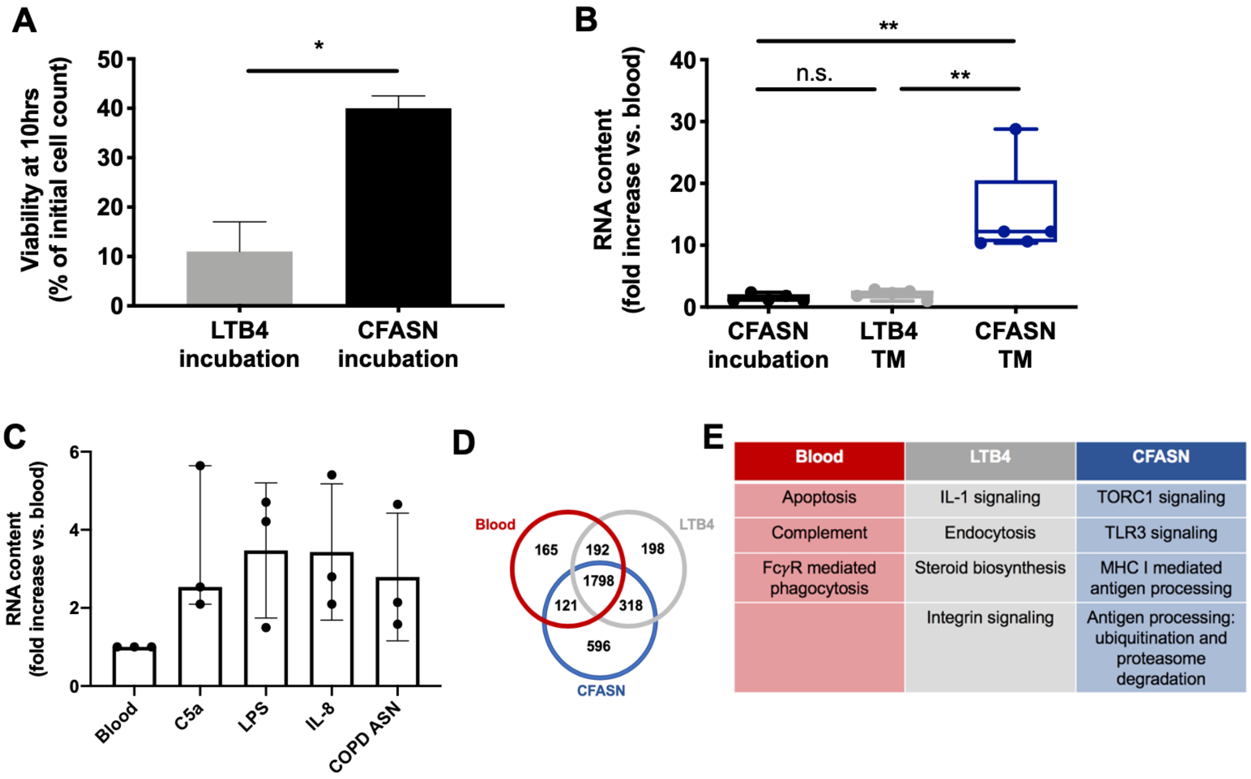

Figure S3 related to Figure 2. Incubation in CFASN increases neutrophil lifespan, but fails to recapitulate the transcriptional burst. Neutrophils isolated from blood of healthy donors were incubated in LTB4 or CFASN for 10 hours and analyzed for viability **(A)**. Neutrophils incubated in CFASN, or transmigrated in LTB4 or CFASN **(B)**, or C5a, LPS, IL-8 or COPD ASN **(C)** for 10 hours were analyzed for their total RNA content. Data are shown as median and interquartile range and were analyzed using the Wilcoxon matched-pairs signed rank test, *$p<0.05$, **$p<0.01$. **(D)** Blood and neutrophils transmigrated *in vitro* for 10 hours (N=3) were lysed and the extracted proteins were analyzed by LC-MS untargeted proteomics. Proteins present in at least two repeats were used to generate the Venn diagram, while unique proteins for each condition were used to determine pathway enrichment by GSEA .**(E)** Pathways listed showed an FDR less than 5% and a p-value less than 0.01.

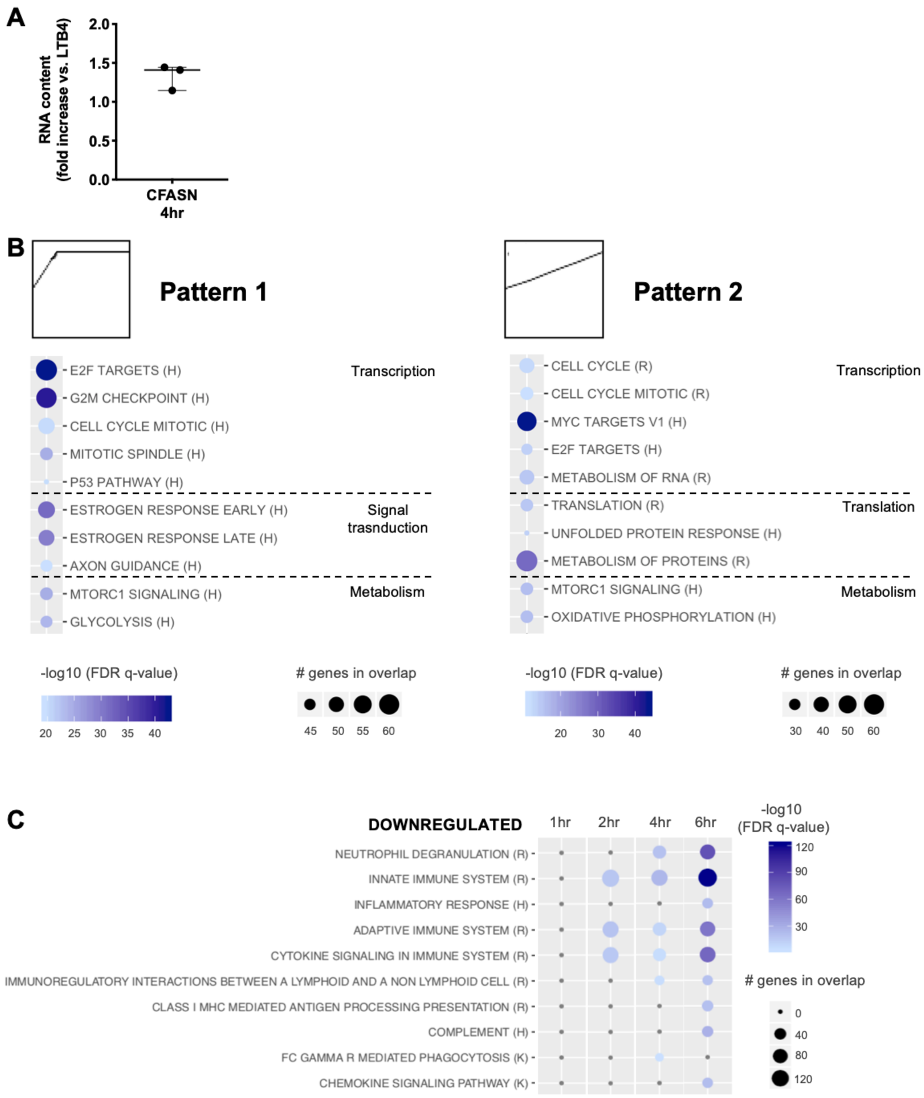

Figure S4 related to Figure 3. Pathway analysis of the GRIM phenotype development. Neutrophils were transmigrated towards CFASN and their transcriptome was analyzed at 1, 2, 4, and 6 hours post-transmigration compared to matched blood (N=3). (**A**) Comparison of total RNA content, measured by bioanalyzer, between CFASN and LTB4 transmigrated neutrophils at 4hr post-transmigration. Upregulated genes were analyzed by STEM and two patterns of gene upregulation over time were identified **(B)**. Pathway enrichment analysis was performed by GSEA (FDR q-value <5%) for upregulated **(B)** and downregulated genes **(C)**.

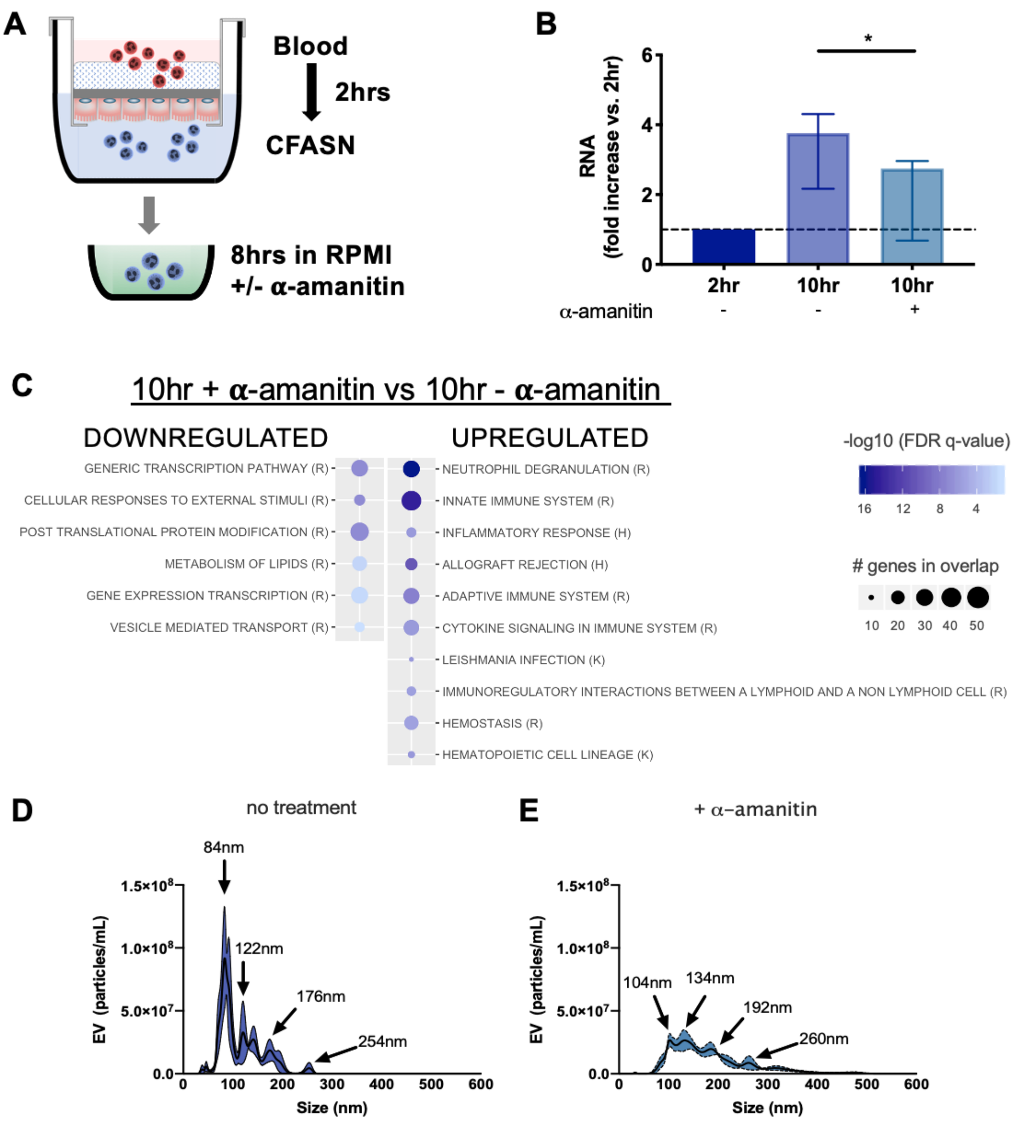

Figure S5 related to Figure 4. Blockade of *de novo* transcription modulates airway neutrophil phenotype in the CF microenvironment. (A) Neutrophils were transmigrated *in vitro* for 2 hours towards CFASN and subsequently incubated for 8 hours with or without the transcriptional blocker α-amanitin. CFASN transmigrated neutrophils treated with α-amanitin showed reduced amounts of total RNA content **(B)**. **(C)** Comparison of neutrophil treated with the transcriptional blocker to those not treated showed a shift in their transcriptional profile upon treatment. Downregulated and upregulated genes analyzed by GSEA using hallmark (H), reactome (R), and Kegg (K) datasets. Data are shown as median and interquartile range and were analyzed using the Wilcoxon matched-pairs signed rank test, *$p<0.05$. Extracellular vesicle concentration and size were determined using the Nanosight NS3000 for neutrophil not treated **(D)** and treated **(E)** with α-amanitin.
